# How do adults and teens with self-declared Autism Spectrum Disorder experience eye contact? A qualitative analysis of first-hand accounts

**DOI:** 10.1371/journal.pone.0188446

**Published:** 2017-11-28

**Authors:** Dominic A. Trevisan, Nicole Roberts, Cathy Lin, Elina Birmingham

**Affiliations:** 1 Faculty of Education, Simon Fraser University, Burnaby, British Columbia, Canada; 2 School of Audiology & Speech Sciences, University of British Columbia, Vancouver, British Columbia, Canada; Harvard Medical School, UNITED STATES

## Abstract

A tendency to avoid eye contact is an early indicator of Autism Spectrum Disorder (ASD), and difficulties with eye contact often persist throughout the lifespan. Eye contact difficulties may underlie social cognitive deficits in ASD, and can create significant social and occupational barriers. Thus, this topic has received substantial research and clinical attention. In this study, we used qualitative methods to analyze self-reported experiences with eye contact as described by teens and adults with self-declared ASD. Results suggest people with a self- declared ASD diagnosis experience adverse emotional and physiological reactions, feelings of being invaded, and sensory overload while making eye contact, in addition to difficulties understanding social nuances, and difficulties receiving and sending nonverbal information. Some data support existing mindblindness frameworks, and hyperarousal or hypoarousal theories of eye contact, but we also present novel findings unaccounted for by existing frameworks. Additionally, we highlight innovative strategies people with self-declared ASD have devised to overcome or cope with their eye contact difficulties.

## Introduction

An old English proverb proclaims, “The eyes are the window to the soul.” For astute observers, eyes hold a wealth of information about a person’s emotions, mental states [1], identity [2], and focus of attention [3]. In a matter of minutes, reciprocal eye contact can invoke passionate feelings of love and affection between complete strangers [4], and eye contact between an infant and caregiver represents one of the earliest, most potent, social interactions necessary for developing attachment, and for the development of relationship maturity and emotional competencies later in life [5]. Surely, reciprocal eye gaze is one of the most powerful and meaningful social interactions humans share [6]. For most of us eye contact comes naturally and effortlessly. So why then, is eye contact so difficult, distressing, and sometimes impossible for certain people—perhaps especially those with Autism Spectrum Disorder (ASD)?

Autism Spectrum Disorder (ASD) is a neurodevelopmental disorder, with onset occurring around the age of two years, and persisting throughout the lifespan. ASD is characterized by the presence of multiple symptoms from each of two broad sets of criteria; 1) deficits in social communication and social interactions, and 2) Restricted, repetitive patterns of behavior [7]. Concerning the first set of criteria, communication difficulties extend to both verbal and nonverbal domains, ranging from reciprocity, empathetic gestures, facial expressions, eye contact, mannerisms, and the social use of language (e.g., turn-taking in conversations). An inhibited tendency to look at and follow the eyes of an adult are some of the earliest indicators of an impending ASD diagnosis ([8–12] c.f.[13]), and atypical eye contact is reported to persist throughout childhood and into adulthood for many individuals with ASD [14]. Because the eyes hold a major source of communicative information, avoiding or ignoring the eyes of others can result in repeated missed social and emotional learning opportunities during early childhood that compound to adversely impact social cognitive development [15]. Later in life, atypical eye contact can cause significant barriers and challenges for people with ASD for the purposes of regulating real-world social interactions [16].

### The theories

Several existing models attempt to explain atypical eye contact in ASD [14]. The *hyperarousal/gaze aversion model* suggests that looking at the eyes of others is aversive, and that people with ASD avoid eye contact and faces to prevent negative affective arousal [14, 17–19]. This model would predict that people with ASD actively avoid eye contact rather than passively omitting it [14]. In support of this model, Dalton and colleagues [17] observed that looking at the eyes of faces elicits over-activation in limbic regions such as the amygdala in ASD, which the authors inferred to reflect hyperarousal in response to eye contact. Another study observed no group differences in activation of subcortical face processing regions in ASD participants when free-viewing face stimuli, but when gaze was constrained to the eye region of the faces, the ASD group show significantly higher activation relative to control participants [20]. A related study demonstrated that children with ASD show higher levels of arousal (as measured by skin conductance response) when viewing face stimuli with direct gaze compared to averted gaze stimuli, while neurotypical children’s arousal responses were not differentiated by the direct and averted gaze conditions [21]. Despite these and other findings supporting the hyperarousal model [18, 21–22], it may not explain all instances of eye contact disturbance in ASD [14,23], suggesting that other mechanisms are at play [24].

For instance, the *hypoarousal*/*social motivation* model suggests that the amygdala fails to prioritize social information in the environment, and as a result stimuli like faces and eyes are not preferentially attended to in ASD [25–28]. This account suggests that social information is less intrinsically rewarding to individuals with ASD [29]. In typical development, eye contact is thought to have intrinsic reward value such that repeated experiences of attaching positive social experiences co-occurring with eye contact will lead to a conditioned motivation to seek out eyes. Dawson and colleagues argue that due to hypo-activation of the amygdala, people with ASD do not pair reward value with eye contact and thus are ambivalent towards others’ eyes [30]. For example, during a face perception task, participants with ASD showed significant reduction in activation of face-processing networks such as the fusiform gyrus and amygdala [31]. A similar study found reduced amygdala activation during a face-viewing task in ASD compared to control participants, and no change in amygdala activation in the ASD group when fixations were experimentally directed to the eye region [32]. However, this study also found reduced activation in the fusiform gyri (FFG) of ASD participants during the free viewing task, but that FFG activation was “normalized” to the level of the control participants when visual scanpaths to the eye region were constrained, suggesting some components of social brain function can be improved or normalized in ASD when forcing direct eye contact. Another compelling study used eye-tracking methods in two-year olds with and without ASD to examine group differences in eye contact in various conditions [33]. When children with ASD were cued to look into the eye regions of adults, they did not show aversion to eyes by looking away sooner than the neurotypical participants. However, when they were not cued to look into the eyes, the ASD group showed diminished eye contact, suggesting a relative indifference to eyes in support of the hypoarousal model. As with the hyperarousal/gaze aversion model, the hypoarousal/social motivation model has received mixed support [14,23–24,34–35].

The *mindblindness framework* of ASD [36] also has strong implications for eye contact abnormalities in ASD. This theory suggests that individuals with ASD are born without an innate module that promotes sharing attentional states with others based on information from their eye gaze (eye direction detector, EDD) [36]. This shared attention mechanism (SAM) normally allows information about states of “seeing” (from the eyes of others) to be used to infer mental states (Theory of Mind Mechanism ToMM). Deficient functioning of one or more of these modules would reduce the degree to which individuals with ASD attend to others’ eyes to determine the intentions and mental states of others. Being a difficult model to test directly (although see [37]), evidence for this theory is mainly indirect, from findings of weaker or absent performance on theory of mind tasks [38] and reduced levels of joint attention [39–40] in ASD.

Importantly, these three theories of atypical eye contact in ASD make very different predictions about how people with ASD will experience eye contact. The *hyperarousal/gaze aversion* model predicts that individuals with ASD will report experiencing aversive reactions to making eye contact with others; the *hypoarousal/social motivation* hypothesis predicts that people with ASD will report either not being interested in making eye contact or failing to see the importance of making eye contact with others. The *Mindblindness framework* would best align with reports of not understanding of the meaning portrayed by the eyes, specifically in terms of their ability to reveal information about the intentions, beliefs, and emotional states of others.

### The present study

Experimental and observational research has provided important information about eye contact abnormalities in ASD. Yet, our understanding of atypical eye contact in ASD remains incomplete. The purpose of this study was to explore the subjective experiences of people with self-declared ASD to provide needed ecological validity to our understanding of atypical eye contact in this population. Autobiographical accounts of notable authors with ASD have commented on the reasons behind their own eye contact difficulties [41–42]—suggesting that the research community may have much to learn from self-reported experiences. Furthermore, the theories described earlier were derived from tightly controlled experimental studies and from observational evidence. Yet, the conditions in which eye gaze responses are experienced and measured in controlled laboratory settings could be quite different than how eye contact is used and experienced in real-world settings—particularly since in the latter, the eyes being looked at belong to a real person looking back at the person with ASD. Thus, it is helpful to examine the lived experiences of eye contact in people with ASD to corroborate or challenge our theoretical assumptions, and perhaps extend existing models or generate new ideas that could be empirically tested.

Increasingly, researchers are becoming aware of the need to incorporate the voices of people with ASD into research [43–44] to aid our understanding of this complex disorder. However, traditional qualitative methods like face-to-face interviewing present unique challenges for the ASD population, considering the social communication difficulties and social anxiety common to this disorder [43,45]. Speaking as a person diagnosed with Asperger’s syndrome, Singer has lamented that face-to-face interactions are difficult and awkward for people on the autism spectrum, but when unrestricted by the social intricacies of conversational turn-taking, body language, and eye contact, some people with ASD can communicate effectively and eloquently online [46]. Thus, we chose to analyze accounts of people with self-declared ASD describing their experiences with eye contact on the Internet. An initial search found that many people with ASD participate in chat rooms and popular websites to share their life experiences and perspectives. Two websites in particular—YouTube.com and WrongPlanet.net (see Methods section)—provided a vast dataset of people with self-proclaimed ASD diagnoses describing their experiences with eye contact.

A central goal of qualitative research is to examine how a particular group of people understand and interpret their social reality in naturalistic contexts [47]. That is to say, qualitative research is especially useful for exploring how people make sense of a phenomenon of interest, by exploring their actions, beliefs, perspectives, and experiences [47]. We started the study with one overarching question, *“How do people with self-declared ASD experience eye contact?”* This question aimed to explore the thoughts, feelings and bodily sensations that are experienced during face-to-face interactions involving eye contact. This question was broad and vague by intention, as we had little expectations as to what we would find and wished to be as inclusive as possible. While we did not set out to validate the various theories of atypical eye contact in ASD, nor was our study designed to do so, we were interested in how each theory might be borne out in the lived experiences of individuals with ASD, or if new information might emerge from the data.

As the data collection process unfolded, we observed some discussion about eye contact that we deemed important, but did not directly answer our initial research question. Starting with such a broad open-ended question helped spur other research questions and guide our analyses. For example, many people discussed their views on social expectations about when and how to use eye contact in various social interactions and settings. In addition, as many of its members use WrongPlanet.net as a resource for peer support, some people used the forum section of the website to ask others for advice on how to cope with eye contact difficulties, or tips on how to get better at it. Thus, to accommodate other important information, we created two additional research questions: *“What beliefs do people with self-declared ASD hold about the societal and cultural norms of eye contact?”* and *“What strategies do people with self-declared ASD use to improve their eye contact*, *or compensate for difficulties with eye contact?”* Thus, our first research question was developed before investigating the data. The latter two research questions were derived from the data.

## Methods

### Participants

This study analyzed secondhand data from publicly accessible Internet sites; Youtube.com and Wrongplanet.net. It would have been impossible to seek permission from individuals who participate in these sites as their personally identifying information is not available. Thus, all such data reported in our article is completely anonymous. YouTube policy requires all uploaders to agree to release rights to their videos, making them publicly available to be viewed, used, and redistributed in anyway YouTube users wish. All forums on WrongPlanet are publicly accessible. Approval of this study’s methodology (application # 2016s0014) was granted by the Research Ethics Board (REB) of Simon Fraser University, who deemed the study "Minimal Risk." We complied with the terms of service for both websites.

We searched for videos of people with ASD on YouTube describing their own personal experiences with eye contact using the search terms “autism” and “eye contact.” Although we could not verify diagnosis, we only accepted YouTube videos in which the individual disclosed a diagnosis of Autistic Disorder, Asperger’s Syndrome, or Autism Spectrum Disorder. In total, 10 videos on YouTube were deemed eligible for analysis, and subsequently transcribed for analysis. Text within each transcription was analyzed only if it directly answered one of the three research questions. Any independent unit of text from the entire dataset that was deemed relevant is referred to henceforth as a “meaning unit.” The videos contributed to 75 separate meaning units, included as part of Table 1, for an average of 7.5 meaning units per individual.

**Table 1:** How do people with ASD experience eye contact?

Main Themes	Sub-themes	Number of Meaning Units	Percentage of Total Meaning Units from Research Question #1
**Adverse Reactions**	*Fear/Anxiety*	16	3.34%
	*Physiological Reactions*	20	4.18%
	*Pain*	22	4.59%
	*Threat Response*	24	5.01%
**Invasion**	*Violation*	54	11.27%
	*Fear of Conveying Private Information*	15	3.13%
	*Intimacy*	56	11.69%
**Sensory Overload**	*Audiovisual Integration*	81	16.91%
	*Energy Exertion*	13	2.71%
**Social Nuances**	*Feels Unnatural*	32	6.68%
	*Confusion about Appropriate Use of Eye Contact*	53	11.06%
	*Self-Consciousness & Embarrassment*	31	6.47%
**Nonverbal Communication**	*Difficulties Reading Information from the Eyes*	27	5.64%
	*Inaccurate Nonverbal Sending*	35	7.31%

We also analyzed texts from discussion forums on Wrongplanet.net, an online community of over 80,000 registered users who identify as having ASD. We typed in the keywords “Eye Contact” in the WrongPlanet search query and found an astonishing 1424 separate posts from 62 forum threads related to eye contact on Wrongplanet.net as of April, 2016. These 1424 independent posts were contributed by 768 independent users. Evidently, this topic is of significant interest to the ASD population. In total, 623 separate Wrongplanet meaning units were coded and categorized according to theme and subtheme in Tables 1–3 along with the YouTube data. These 623 meaning units were contributed by 354 separate Wrongplanet users, yielding an average of 1.76 meaning units per user. The vast majority of these 354 posters only contributed one meaning unit that was analyzed (n = 249). Combining YouTube and Wrongplanet data, Tables 1–3 include 698 separate meaning units from 364 distinct individuals.

### Procedure

After searching for and locating relevant videos and texts from YouTube and WrongPlanet, videos were transcribed and uploaded into the qualitative analysis software, NVivo [48]. Relevant webpages from Wrongplanet were converted into.pdf files using NVivo’s nCapture add-on before being uploaded into NVivo as readable text.

### Data analysis

We analyzed data using *qualitative content analysis* methods, which is appropriate for analysis of recorded human communications including books, web pages, magazines, speeches, newspapers, etcetera [49]. Texts that were deemed relevant to the research questions were copied as a “meaning unit” and organized into categories using an open coding method—the process of breaking down, examining, comparing, conceptualizing and categorizing data [50] to form the main themes comprised by their subthemes. Texts were only coded if they answered one of the three research questions described previously. Each category was carefully defined within NVivo as they were created, and adjusted throughout the coding process. During and after each coding session, descriptions of—and rationales for—what was accomplished throughout each session were described in a journal and constantly referenced during subsequent coding sessions. Throughout the coding process, the lead author (DAT) regularly met with the other co-authors on this paper to discuss progress, brainstorm ideas, and deliberate problems. The coding process initially resulted in 35 preliminary categories. After the coding process was finished, DAT met with the second author (NR) on multiple occasions to revise the definitions of the categories, merge highly similar categories, and delete categories that were deemed to be flawed, problematic, or trivial in comparison to the more prominent categories. Through this process, the categories were organized and re-organized into a final agreed upon 8 themes, each containing 2 to 7 subthemes. The themes and subthemes are described in the Results section, and summarized in Tables 1–3.

## Results

### Research question #1. How do people with self-declared ASD experience eye contact?

Our analysis of this particular research question revealed 5 major themes (bolded) and 14 subthemes (italicized), described below and found in Table 1. Direct quotations (italicized and indented) are incorporated throughout to exemplify each subtheme. Additional quotations for each subtheme can be found in S1 Appendix.

#### 1) Adverse reactions

This theme includes subthemes related to negative emotional and physiological reactions experienced in response to eye contact, and subthemes related to pain, and threat responses. Data in this theme provide support for the basic premise of the hyperarousal model, which predicts that people with ASD will experience affective arousal in response to eye contact.

*Fear/Anxiety*. The most common emotions reported in the data included anxiety, panic, fear.

Making eye contact feels sort of like the first breath one takes under water using scuba gear, where there’s this moment of panic as your body says, ‘No, no, you’ll drown!’

Another said,

I get nervous but not like scared nervous just like um…like a shaky kind of nervous

*Physiological Reactions*. Negative physiological reactions in response to eye contact ranged from feelings of dizziness, lightheadedness, headaches, watery or tired eyes, increased heart rate, nausea, tremors, and overheating. Several people compared the sensations of eye contact to be similar to that of “staring into the sun.”

If I am forced to make eye contact, my body becomes tense, my skin tingles, my jawline becomes somewhat numb.

Another person said,

Eye contact just makes my stomach twist and makes me feel like I want to vomit.

Another said,

I’d say it always causes a lot of discomfort and stress, psychological and physical (Tremors, stuttering, sometimes headaches).

*Pain*. Some people (22 meaning units) described eye contact as being “painful” or otherwise “uncomfortable.”

Eye contact is sort of like getting shocked or something; it is very unpleasant and almost hurts, and if I am forced to do it for any length of time I get increasingly panicked.

Another said,

It’s like biting into a really sour lemon or licking the end of a battery. The feeling of tiny creepy crawlies shimmering under your skin making you cringe. Butterflies in your tummy trying to escape out through your head.

Others were quite blunt, stating,

Eye contact is physically painful

or,

It hurts like hell.

*Threat Response*. Several people (24 meaning units) described perceptions of others looking at them as threatening, triggering a “fight or flight” response.

[Eye contact] makes me feel like prey that’s been spotted by a predator.

Another said,

For me… eye contact triggers a fight or flight response so strong that it overrides everything else, including my better judgment of the situation.

#### 2) Invasion

Another important theme was feelings of being invaded or violated when being looked at in the eyes. Subthemes included *violation*, *fear of conveying private information*, *and intimacy*.

*Violation*. Many people (54 meaning units) described feelings of being violated when being looked at in the eyes, making them feel “overexposed,” fearing that others could “peer into their souls.”

It makes me feel naked, exposed. Weak if you will. It’s very uncomfortable.

One person likened being looked at in the eyes to that of being,

…raped on a spiritual level.

Someone else said,

If I find it too hard to give eye contact, it sometimes relates to not being comfortable making that connection with people. Forcing me to look at someone is forcing an intimacy that does, indeed, have a tone of violation.

*Fear of conveying private information*. Perhaps one reason eye contact was perceived as invasive to some is because it felt like an intrusion of privacy, or even unwanted solicitation of private information.

I talk so much with my eyes–people can read what I’m thinking and feeling, regardless of my body language and voice, just by looking in my eyes…I don’t like people knowing more about me than what I’m saying.

Relatedly, these feelings of conveying private information may come from a sense of shame, or fear of judgment.

I’m bad at ‘bluffing’: When I’m tired [or depressed]. I think that shows up in my eyes and is hard to hide…For as pleasant as I try to be, I know that to some extent, people can see through that and understand that I do have issues, whether it’s my social awkwardness, depression, etc., and my eyes are the express route to that understanding, which I do find intimidating, being a person who is private with his feelings in the first place and who does fear some degree of judgment.

*Intimacy*. Many people (56 meaning units) alluded to a belief that eye contact was considered a very intimate experience that is only appropriate with loved ones.

Maintaining eye contact feels waaaayyy [too] personal and intimate, and with someone I don’t know very well that can feel downright creepy.

Similarly, someone stated,

Eye contact is in inherently uncomfortable thing for me, that I can only achieve with those whom I have a degree of intimacy or trust with.

#### 3) Sensory overload

Perhaps the most common context in which people typically make eye contact is during face-to-face conversations. In this context, many people described an inability to listen to another person while making eye contact at the same time. We interpreted statements like this as representing “sensory overload” [43], due to difficulties processing and integrating visual and auditory information simultaneously. Feelings of ‘overload’ were supported by other reports relating to how tiring and effortful it is to maintain eye contact. Two subthemes emerged from this theme, including *energy exertion* and *audiovisual integration*.

*Energy Exertion*. Some people (13 meaning units) described eye contact as being exhausting, requiring an enormous exertion of energy.

For me [eye contact] feels like I’m using up a lot of energy. The longest I can stare at someone in the eye is from less than 2 to 6 seconds at the most. Then it gets tiring.

Another said that eye contact,

…saps my energy [from] my core like being hypnotized by a cold-blooded energy sucking vampire.

*Audiovisual Integration*. Many (81 meaning units) described difficulties processing audio and visual input simultaneously.

I can’t concentrate while making eye contact, particularly if I need to listen to what the other person is saying to me. It’s like I need to shut off the visual input in order to completely process the aural input.

Several people suggested that there are too many distracting features in the face to focus on anything else. Others said their mind just “shuts down” while making eye contact. One person noted a frustrating irony:

[If I] …don’t make eye contact…I’m able to concentrate on the conversation but people think I’m not [paying attention. If I do] make eye contact, I’m unable to concentrate on the conversation but people think I’m more attentive.

Besides concentrating on their conversational partner’s verbal messages, several meaning units indicated that eye contact interfered with their ability to generate thoughts and responses.

Eye contact interrupts the flow of my thoughts. It is as if the eye contact itself becomes the primary thing of which I am cognizant and thinking takes a back seat. Depending on the amount of mental effort required to maintain the conversation, eye contact can be very disruptive.

#### 4) Social nuances

Whereas most neurotypicals engage in eye contact naturally and effortlessly, the opposite was noted for many people with ASD, who described how unnatural and awkward it feels for them. Thus, there was a steep learning curve for some as they struggled to understand the subtle nuances of appropriate use of eye contact. Many emphasized that eye contact does not necessarily cause them adverse reactions like it does for others on the autism spectrum, but that they simply avoid it out of concern of doing it “incorrectly.” A common challenge was understanding how long to maintain eye contact before looking away, and back again during conversations.

*Confusion about Appropriate use of Eye Contact*. Several people (53 meaning units) expressed confusion about how to use eye contact in socially expected manners.

My big problem for a very long time, and probably still, is to determine how much eye contact is appropriate.

Several people were told they “stare” or have an “unrelenting gaze.”

…I’m a starer and always have been…and [neurotypicals] have been dissing on me for as long as I can remember about it.

Others expressed confusion about the nuances of eye contact in various contexts, such as passing others on the street or in a hallway, within a large group or at a party, or for the purposes of flirting.

Isn’t that flirting…if you make eye contact without talking? I get really embarrassed and I just don’t know what to do.

*Feels unnatural*. Some (22 meaning units) emphasized just how unnatural and awkward eye contact feels to them.

I don’t do much eye contact because it doesn’t come naturally to me. I don’t really know the appropriate timing, and I feel at risk of staring for too long, which could be taken as threat-stare or a sexual stare.

Similarly, someone stated,

I’m always afraid that I’ll give eye contact incorrectly. Sometimes women smile at me and give eye contact and I don’t know how to reciprocate other than by smiling awkwardly and looking away from them.

*Self-consciousness & Embarrassment*. Uncertainties about using eye contact correctly lead many (31 meaning units) to describe feelings of self-consciousness and embarrassment that interfered with their social interactions.

The internal monologue starts up about whether or not I should keep looking into someone’s eyes, or if I should look away [be]cause I’m creeping them out, or whether I need to look at them because they now think I’m not paying attention…(which I’m not, because I’m too busy thinking about eye contact).

Similarly, others found eye contact embarrassing.

I find eye contact embarrassing unless I know the person/people well. It makes me feel flustered and I start blushing

#### 5) Nonverbal communication

An important theme described in the data was a tendency to avoid eye contact due to difficulties interpreting emotional information around the eyes. Subthemes included, *difficulties reading information from the eyes*, and *inaccurate nonverbal sending*.

Difficulties Reading Information from the Eyes.

Some people (27 meaning units) reasoned that they may not be drawn to the eyes of others, simply because they are not able to extract useful nonverbal information from the eye region.

My lack of eye-contact started off as the result of confusing social cues. I did not want to look at people who communicated very heavily with their eyes because it was difficult for me to understand.

Another wrote,

I can see that people are using it to communicate something to me, but I can't tell what they're saying.

Similarly, others noted that they need other information to make mental state inferences.

…when I just look at the eyes, I don’t understand the emotion the other person is exhibiting. I need lots of other visual clues–posture, tilt of head, tone of voice. With eyes and mouth alone I really don’t get it. It’s like there is nothing there…

*Inaccurate Nonverbal Sending*. In addition to reading nonverbal cues, thirty-five meaning units referred to difficulties *sending* nonverbal cues. It was a common worry of individuals that they were giving wrong impressions, or conveying inaccurate nonverbal communicative information to others during face-to-face interactions. For instance, recognizing that he was fairly non-expressive, one individual said that his peers often,

…[mis]interpreted my blank facial expression/lack of eye contact as either sadness or boredom with them.

Another said,

If people don’t see my face, then I can’t send them unintentional signals…So whenever I’m talking to people that know me, I try my absolute hardest to make sure they don’t see my face.

One person frustratingly exclaimed,

If I don’t participate in [eye contact] for long periods of time, people are going to tell me either I am shy, submissive, or I’m being rude by looking away. Or they would assume I’m being shifty by looking away and I don’t know why that is.

Table 1 describes the themes, subthemes, and number of meaning units that resulted from Research Question #1.

### Research question #2. What beliefs do people with Self-Declared ASD hold about the societal and cultural norms of eye contact?

#### 6) Society & culture

For this theme, there was some disagreement—some emphasized the *importance* of eye contact, whereas others emphasized the *lack of importance* of eye contact. An additional subtheme related to *neurodiversity advocacy*.

*Importance*. Some people (21 meaning units) recognized the importance of eye contact for the purposes of succeeding in job interviews, interacting with colleagues and customers, and for dating and interacting with friends.

Eye contact is one of the most uncomfortable things in the world to me. And because I work in sales I have to do it a lot and I HATE IT!

Some begrudgingly admitted that, despite the discomfort it causes, they simply have to do it.

…it’s what I have to do to get by in this [neurotypical] world!

Another lamented,

To put it bluntly, I suck it up and just deal with it. No one goes through life without experiencing discomfort or unappealing things.

Another noted that mastering eye contact has made his life easier.

It’s useful to have people think that I'm honest and interested in what they’re saying. It makes life run more smoothly.

*Lack of importance*. A few people (6 meaning units) expressed disdain for the social practice of eye contact, failing to see the value of this social convention.

It just doesn’t make sense. Why would you want someone to look into your eyes? Unless maybe [you’re] in love with them but otherwise…

Another said,

I never understood the need for it. People can hear me just fine, and I can hear them without having to look into their eyes. I’ve no interest in looking people in the eyes just because it’s some unspoken social rule.

*Neurodiversity advocacy*. Some people (29 meaning units) described experiences of strong pressures from caregivers or therapists to make eye contact when they were children, and described them as traumatic experiences.

My mother would grab my face and yell at me to look at her when she was talking. I would panic and start to sense that darkness was closing in all around my field of vision. My heart would race and I would feel like I might vomit. I would shake.

On this topic, individuals were all but unanimously critical of such interventions. One person angrily described such interventions as “neurotypical tyranny”—a misguided attempt to “normalize” people with ASD for the comfort of neurotypicals. Some advocated for increased awareness of “neurodiversity,” and the belief that people with ASD should not have to engage in eye contact just to conform with societal expectations.

…it is absurd, prejudicial, and unfair. I am, to some extent, physically unable to [make eye contact]–and I was made to suffer for that…the bigots need to stop forcing us to be like them, and accept who and what we are.

One person took an opportunity to advocate for children on the autism spectrum who may not be able to verbally articulate feelings of discomfort.

[Eye contact] can be about as comfortable as standing naked outside in zero degree weather near the entrance of a very busy shopping mall….I’m saying this for the autistic three-year-old girls of the world who can’t articulate those feelings.

Table 2 describes the themes, subthemes, and number of meaning units that resulted from Research Question #2.

**Table 2:** What beliefs do people with self-declared ASD hold about the societal and cultural norms of eye contact?

Main Theme	Sub-themes	Number of Meaning Units	Percentage of Total Meaning Units from Research Question #2
**Society & Culture**	*Importance*	21	37.50%
	*Lack of Importance*	6	10.71%
	*Neurodiversity Advocacy*	29	51.79%

### Research question #3. What Strategies do People with Self-Declared ASD use to Improve their Eye Contact, or Compensate for Difficulties with Eye Contact?

#### 7) Strategies to improve eye contact

Fifty-one separate meaning units contained strategies for *improving* eye contact–generally describing ways to become more comfortable with using eye contact or getting better at using it appropriately.

*Exposure & Practice*. Some people (22 meaning units), discussed concepts of “exposure therapy,” whereby they began by practicing brief glances to others’ eyes at first, and gradually becoming more and more comfortable over time. Interestingly, many described the usefulness of practicing first with photographs or mirrors before working up to family and close friends, before becoming comfortable making eye contact with strangers.

As one person suggested,

Practice on someone you feel the most comfortable with–even a relative. You may not be able to maintain it and it can be scary at first. Try for a few seconds at a time. Once you can do that, practice on other people.

Others mentioned that eye contact was much easier to practice with pets or other animals before working up to human eyes.

It is generally easier to make eye contact with animals than with humans, that might be a less distressing first step and making eye contact with humans might be the next step.

*Barrier*. Some people (9 meaning units) suggested using a barrier, such as sunglasses, seeing glasses or contact lenses made eye contact more bearable.

You’re about to learn a major secret of mine. [My glasses] don’t magnify or anything…they’re not reading glasses…wearing these glasses it’s like …I can look at a movie screen and see the characters’ eyes and everything… glasses are perhaps the biggest thing that really helped me with eye contact. Because you know there’s this extra barrier.

*Observation*. Some people (4 meaning units) said they observe how neurotypicals use eye contact and try to mimic their behaviors.

Another thing you want to work on is observation. You want to notice what other people do…and then you can try to mimic that.

*Counting*. This strategy was not necessarily for becoming more comfortable with eye contact, but to avoid “staring.” It involved looking away periodically (every 2–5 seconds according to different accounts) to break sustained eye contact.

I’ve had to make sure I maintain eye contact for the appropriate length of time. The average American maintains eye contact an average of 3–5 [seconds]…I tap my toes (not my foot) to count out the seconds. If I get to five [seconds], I take a fleeting glance at the wall or at my toes. No one has accused me of staring for months now.

*Mental Distraction*. Several people (8 meaning units) emphasized the importance of working towards eye contact becoming second nature.

I now know one of the important things is not to force yourself to have eye contact and not to think about eye contact. If you think about eye contact, that makes you very self-conscious and awkward, which makes the other person very uncomfortable as well. I realize good eye contact happens rather naturally without being too conscious of it.

*Motivation*. A few people (3 meaning units) recommended finding ways to motivate themselves for becoming better at eye contact. For example, for successful dating, or for getting better at reading people.

No matter how bad you are at it, give yourself a reason to do it. Like you want to show you’re paying attention, or you want to try to read someone’s expressions. Learning to read people helped me a lot since I play poker. Even though it doesn’t come across as friendly.

*Other*. Other strategies (2 meaning units), related to looking out the corner of one’s eyes, or focusing on one eye only, although it was unclear what these strategies were meant to achieve.

I look out the corner of my eyes, in that cute little way.

#### 8) Strategies to compensate for lack of eye contact

As described in 112 separate meaning units, eye contact for some people is so challenging that achieving it is not the goal. This subtheme describes ways to *compensate* for lack of eye contact using less taxing actions. This theme includes 7 subthemes, described below and summarized in Table 3.

**Table 3:** What strategies do people with self-declared ASD use to improve their eye contact, or compensate for difficulties with eye contact?

Main Themes	Sub-themes	Number of Meaning Units	Percentage of Total Meaning Units from Research Question #3
**Improvement**	*Exposure & Practice*	23	13.50%
	*Barrier*	9	5.52%
	*Observation*	3	2.45%
	*Counting*	3	1.84%
	*Mental Distraction*	8	4.91%
	*Motivation*	3	1.84%
	*Other*	2	1.23%
**Compensation**	*Non-eye Fixation*	67	41.10%
	*Verbal Backchanneling*	3	1.84%
	*Nonverbal Backchanneling*	10	6.13%
	*Disclosure*	3	1.84%
	*Body Position*	2	1.23%
	*Blurred Focus*	9	5.52%
	*Strategic Eye Contact*	19	11.04%

*Non-eye fixation*. Easily the most common strategy (67 meaning units) was to look at the face without looking directly at the eyes. Some described looking at the face as a “whole” without focusing on eyes, and many suggested focusing on parts of the face near the eyes such as the nose or mouth.

I usually look at their mouths, especially if they’re talking and it’s moving. If I have to make eye contact with someone I know I look at the bags under their eyes, and then I slide up to meet their eyes and then slide down to eye-bags again. I don’t think people notice it so much if you look somewhere really really close to their eyes.

Another said,

My advice for if eye contact intimidates or scares you like it does me is just look at the whole face. If you look at it as a whole you don’t find it scary (I don’t at least) and it looks like you are making eye contact.

*Verbal backchanneling*. A few people (3 meaning units) said they use verbal cues to indicate attention to the conversation (so that a lack of eye contact doesn’t give a false impression of boredom).

I would say stuff like: really, sweet, I know what you mean, that’s cool…stuff like that to show I am following their sentence.

*Nonverbal backchanneling*. Several people (10 meaning units) suggested using body language (such as facial expressions or nodding) to indicate attention the conversation.

[I] try to compensate by doing lots of appropriate body language, not crossing my arms, smiling and making “active listening” type noises to show I’m listening….People want to know you care; and if you show that then it shouldn’t have to be as didactic as percentage of time looking as opposed to not.

*Disclosure*. A couple people (3 meaning units) suggested simply explaining that they have trouble with eye contact, or offering a cover story.

…if I ever get called out on [my lack of eye contact], I either say "yeah, my ears are blocked at the moment, so I have to look at your mouth" or "I'm the kind of person who needs to look away a little to really concentrate on what you're saying."

*Body position*. Two people (2 meaning units) suggested positioning one’s body in such a way that eye contact is not expected.

How do I avoid it? Simple; don’t sit in front of someone you are talking to. Sit beside them, behind them, or anywhere other than face to face.

*Blurred focus*. Other innovative strategies were introduced (9 meaning units) describing ways to make eye contact more bearable. For example,

I am not sure how to describe this, but I also unfocus my eyes, so I am giving eye contact but for me [it's] a fuzzy, less intense view…

*Strategic Eye Contact*. Others (18 meaning units) identified the critical moments during conversation at which eye contact is most essential, which enabled them to appear more socially competent and reduce the needed amount of eye contact required of them to a manageable amount.

I've found that eye contact is essential when starting a conversation with someone. Once the ball gets rolling, eye contact is not required that much anymore. That's when I do it in short bursts, every once in a while.

Table 3 describes the themes, subthemes, and number of meaning units that resulted from Research Question #3.

## Discussion

The primary objective of this study was to advance our understanding of why people with ASD often have difficulties with eye contact. To this end, we used qualitative methods to explore accounts of people with self-declared ASD who described their lived experiences with eye contact. The themes that emerged were quite varied—relating to negative emotional and physiological reactions, feelings of being invaded, sensory processing issues, difficulties understanding how to use eye contact in socially appropriate manners, and difficulties inferring and sending nonverbal emotional information. As our data were drawn from 364 distinct individuals, it is quite apparent that these individuals make up a highly heterogenous group, with markedly diverse eye contact experiences. As discussed below, some of our data support the *hyperarousal* model, some may provide support for the *hypoarousal* model, and yet others provide support for the *mindblindness* account. In contrast, the data revealed other themes that do not appear to be explained by any of the existing eye contact theories.

While the objective of this study was not to test or disconfirm any particular eye contact theory, it is worth mentioning that our data most strongly support the *hyperarousal* model [17–18]. This finding is best exemplified by our Adverse Reactions theme. The hyperarousal model predicts that eye contact would result in negative affective arousal, and indeed, many subjective experiences categorized in the Adverse Reactions theme described feelings of fear, panic, or anxiety in response to eye contact, as well as physiological responses that are likely indicative of these emotions (e.g., increased heart rate, sweating, shaking, etc.). A number of individuals also reported feelings threatened by eye contact, triggering a “fight or flight” response. Undoubtedly, in some social situations eye contact may actually be threatening (e.g., a “stare-down”), for the purposes of intimidation. However, if an individual consistently interprets others’ eye gaze as threatening, this could represent a hyperarousal response reflecting intense social anxiety, or could potentially reflect a social cognitive deficit, whereby they are misinterpreting neutral (non-threatening) looking behavior of others.

The *hypoarousal* model would predict that faces—particularly the eyes—are less intrinsically interesting to people with ASD [25–29]. Compared to the hyperarousal model there is relatively less support of the hypoarousal model in our dataset. However, there is some data that we speculate may support this model. For example, some individuals said that they are often accused of “staring relentlessly,” which, does not necessarily indicate hypoarousal per se, but may suggest an absence of hyperarousal. Others noted beliefs that eye contact is meaningless or pointless, perhaps indicating a failure to understand or recognize the social information that is exchanged during mutual eye gaze. Given the relative paucity of responses that clearly support the hypoarousal model, it is important to emphasize that our data likely suffers from a selection bias, such that people who experience significant distress as a result of eye contact (i.e., hyperarousal), are more likely to participate in forum discussions about their difficulties with eye contact compared to individuals who are uninterested in others’ eyes or faces in general.

Other data suggested that some individuals with ASD seemed to understand that important information *can* be gained from others’ eyes, but that they were not personally able to detect it (e.g., *Difficulties Reading Information from the Eyes* subtheme). Such an account provides support for the *mindblindness framework* [36]. In this framework individuals with ASD have a dysfunctional social cognitive system, which results in a failure to infer mentalistic information from the eye region of faces. This framework has important connections with the hypoarousal model in that social cognitive deficits and reduced social interest/attention are proposed to be tightly interlinked. The key difference between the two is that of directionality—the *mindblindness framework* suggests that social cognitive deficits (e.g., inability to infer mentalistic information), are primary, which leads to a lack of social interest and reduced social attention (including reduced eye contact). The hypoarousal model, however, suggests that reduced social attention is primary, leading to downstream effects on social cognitive abilities, due in part to a lack of exposure to social information (see Chevallier et al., 2012 for a relevant discussion [29]). Thus, while both models received some support from our data, it is not possible to know from the data how these mechanisms are linked, nor can we comment on the directionality of these links if they exist.

While our data provide support for the prominent theories of atypical eye contact in ASD, a number of interesting findings do not appear to be accounted for by existing theories. For example, we found that for some individuals, eye contact is most difficult when listening to what someone else is saying—inconveniently when eye contact is most socially expected. This may stem from an inability to concentrate on auditory (verbal) information while looking at someone else’s eyes, which may represent sensory overload, i.e., difficulties simultaneously integrating visual and auditory information. This finding is consistent with Jones and colleagues’ (2003) qualitative description of sensory perceptual experiences in ASD [43]. For example, one quote from their data was, “If I’m looking at something and listening to something at the same [time], too much information might come in my eyes and ears at one time…” [43] (p. 116). Similarly, Iarocci and McDonald [51] reviewed several existing autobiographical accounts of sensory processing abnormalities and concluded that a common trend among these accounts was a difficulty processing information from more than one modality at the same time. Empirical research has also demonstrated audiovisual integration deficits in ASD [52–54], which particularly impact speech processing [55]. Our study extends these findings by suggesting that difficulties with audiovisual integration may specifically lead some people with ASD to avoid eye contact with others.

It is difficult to infer how such audiovisual integration difficulties might relate to hyperarousal explanations of atypical eye contact, and this remains an important avenue for future research. If they are linked, it is possible that “sensory overload” as a result of trying to process multiple sensory modalities contributes to hyperarousal. Conversely, hyperarousal in response to eye contact could lead people with ASD to “shut down” or experience a narrowing of attentional and cognitive resources, making it difficult to integrate information across sensory modalities. During social interactions, information from people’s faces is usually dynamic, subtle and fleeting, and in the case of conversation, must be rapidly integrated with auditory information from speech signals. However, most experimental studies that have examined hyperarousal measured it in response to static facial stimuli with the absence of auditory input or other dynamic visual cues from the face. Thus, it is possible that eye contact difficulties in live social interactions stem from different processes (or a combination of processes) than what is observed in the lab. There was one particular quote from our data that illustrates this possibility.

I’ve learned how to read body language and facial expressions really well (I do great on all the tests)…but none of this translated to face-to-face contact. I lose access to that part of my brain. I rely as best I can on word choice and voice tone…

Thus, to capture the full extent of eye contact difficulties in ASD it may be necessary to go beyond the standard laboratory tasks using static images of faces, and instead measure performance in settings that more closely resemble real-world social situations [34,56–57].

Another novel finding from our study concerns descriptions of eye contact as feeling *invasive* or *violating*. Again, it is unclear how this theme may relate to existing theories of eye contact abnormalities in ASD. It is possible that feelings of being invaded may produce negative affect, which in turn contributes to a hyperarousal response. One possible explanation comes from theories of social anxiety. In Bellini’s (2006) model, a temperament associated with a disposition for hyperarousal, social skills deficits, negative peer interactions, and social withdrawal all interact and reinforce each other during development to contribute to social anxiety by the teenage years and beyond [58]. In this model, individuals with a predisposition for hyperarousal are especially prone to being adversely conditioned by negative peer interactions, contributing to heightened social anxiety. In turn, social anxiety will contribute to social withdrawal as a coping mechanism. Over time, perhaps eye contact is conditioned to being perceived as an unwelcome social initiation in socially anxious teens and adults with ASD, that is met with an automatic response of feeling invaded or violated. Future research is needed to explore this idea in more detail.

In addition to exploring how people with ASD *experience* eye contact, we also explored the beliefs people with ASD hold about societal and cultural norms surrounding eye contact. Some people expressed ambivalence or derision about eye contact, seeing little value in this social practice and condemning efforts to improve eye contact as a vain effort to normalize people with ASD for the comfort of neurotypicals. Indeed, several people described such interventions as traumatic experiences. Nevertheless, many recognized that difficulties with eye contact had created personal and professional barriers for them, which motivated them to strategize ways in which they could improve their eye contact behaviors or find ways to compensate for relevant difficulties. To this end, numerous strategies were described and summarized in Table 3.

In therapeutic and educational settings, improving eye contact behaviors in children with ASD has long been a target of intervention, given the assumption that children must first orient towards and attend to the instructor or therapist before meaningful learning can take place [59–60]. To this end, researchers have utilized applied behavior analysis techniques, pivotal response training, peer modeling, role playing and contingent imitation techniques, yielding small to moderate improvements in joint attention and other eye gaze behaviors [59,61–63]. It is important to emphasize that most of these interventions target early childhood, and there are almost no existing evidence-based strategies for improving eye contact in socially motivated teens and adults with ASD. One exception is the Program for the Education and Enrichment of Relational Skills (PEERS), an evidence-based social skills training program for teens and young adults, which discusses the use of eye gaze behaviours for the purposes of flirting, for entering or leaving a group conversation, and how to avoid social blunders such as staring [64]. While useful for articulating the *social nuances* of using eye contact in various social contexts, we could not find any literature on interventions that aim to make eye contact easier for those with teens and adults who experience hyperarousal during eye contact. Given the heterogenous eye contact difficulties identified in this study, it may be necessary to tailor interventions for the specific difficulties of each individual. For example, those who experience hypoarousal may need instruction on social nuances, whereas those who experience hyperarousal may need to develop compensatory strategies. This remains an important area for future investigation, and we hope the ingenious strategies listed in Table 3 will be a useful place to start.

In closing, we wish to highlight several limitations to our study. We could not verify diagnosis of our participants. It is possible that some YouTube and WrongPlanet members self-diagnosed and would not actually meet the criteria for diagnosis. For this reason, it is important to replicate this study in participants with a verified ASD diagnosis to see if their experiences are consistent with that of those reported in the present investigation. In addition, it is unlikely that the experiences described are representative of the entire ASD population, as the individuals who frequent YouTube and WrongPlanet are likely highly interested in discussing aspects of their diagnosis and quite insightful and articulate. In addition, there are likely people with ASD who do not have significant challenges with eye contact and these individuals were generally not represented in our data. Similarly, we suspect that those who have significant negative physical or emotional distress as a result of eye contact (i.e., hyperarousal), may be more inclined to participate in forum discussions than those who experience hypoarousal. As data was collected secondhand, another limitation is that we could not perform “member-checking,” a procedure that involves asking informants whether they agree with the researchers’ interpretations of the data [65]. Finally, it would have been useful to discern from our data whether the themes summarized in this paper are mutually exclusive. In other words, we do not know if the themes represent idiosyncratic experiences within the ASD population, or if the themes represent different ways of describing the same phenomena.

Despite these limitations, the data presented here offer rich description of eye contact experiences in ASD and the strategies reported in the data may have practical implications in therapeutic and educational settings. Indeed, the variety of themes that emerged suggest that eye contact abnormalities in individuals with ASD may stem from a number of mechanisms, and that the mechanisms at play may differ from one individual to the next. The data reported in this study—particularly the strategies theme—further highlight the importance of incorporating the perspectives of individuals with ASD not only in the research process, but also in designing resources, tools, and interventions.

## Supporting information

S1 Checklist Consolidated Criteria for Reporting Qualitative Research Checklist (COREQ). (PDF)

S1 Appendix All meaning units contributing to themes and subthemes. (DOCX)
