# Supplementary material for: How do adults and teens with self-declared Autism Spectrum Disorder experience eye contact? A qualitative analysis of first-hand accounts
Source: PLoS One. 2017 Nov 28;12(11):e0188446. doi: 10.1371/journal.pone.0188446 (PMC5705114; doi:10.1371/journal.pone.0188446)
Supplement: S1 Appendix — (DOCX) [file pone.0188446.s002.docx]

**S1 Appendix. All Meaning Units Contributing to Themes and Subthemes**

**1. Adverse Reactions**

***1.1 Fear/Anxiety***

My advice for if eye contact intimidates or scares you like it does me is just look at the whole face. If you look at it as a whole you don't find it scary (I don't at least) and it looks like you are making eye contact

It was rather painful, and I don't know if it was strictly anxiety

I get this weird surge of anxiety in the front of my head and I have to look down

anxiety and panic

I lose my concentration and feel nervous

making eye contact feels sort of like the first breath one takes under water using scuba gear, where there's this moment of panic as your body says, 'No, no, you'll drown!'

But when I had and have a reaction, it's real fear to meet someone's eyes. I have no idea why.

eye contact is sort of like getting shocked or something; it is very unpleasant and almost hurts, and if I am forced to do it for any length of time I get increasingly panicked

my mother would grab my face and yell at me to look at her when she was talking. i would panic and start to to sense that darkness was closing in all around my field of vision. my heart would race and i would feel like i might vomit. i would shake. my mother and the things around me looked 2-dimensional. all of the speech and other noises started to sound farther and farther away. i think this was fueled by fear/adrenaline?

lack-of-eye-contact became the product of shyness and social anxiety

To be quite honest, I do get nervous when I make eye contact sometimes. This is especially true when someone has something serious to say to me.

I use eye contact when I stare at a guy I like from across the room. Yet when I finally talk to them I make little to no eye contact out of nervousness and I experience a feeling of severe awkwardness.

I try to make myself do it sometimes, but I feel anxiety in the pit of my stomach and have to look away

makes me anxious - am i staring too much and seem creepy? am i not making enough contact and seem "shifty"

It makes me feel awkward and uncomfortable

I get nervous but not like scared nervous just like um... like a shaky kind of nervous

***1.2 Physiological Reactions***

It's like all those feelings consolidating into a tight feeling across your chest and shoulders, hairs on the back of your neck standing up

I find myself forgetting to blink and also beginning to overheat

Sometimes, I feel sorta light headed. Not dizzy, just light headed

1. Burning + auditory pulsating 2. Combinations of the following 3. Just burning or pulsating 4. Headaches 5. watery eyes/tearing 6. increased light sensitivity 7. Nothing

I also force myself to meet someone's eyes in conversation - and the whole time my heart is pounding

its almost painful to look into someones eyes, mine tear up and I get faint.

as to how it feels: it makes my heart beat. it makes me sweat, and my natural instinct is only to look people in the eye when i want them to back down. it's an agressive behaviour on my part, so when people keep up the gaze i feel intimidated and start panicking

my mother would grab my face and yell at me to look at her when she was talking. i would panic and start to to sense that darkness was closing in all around my field of vision. my heart would race and i would feel like i might vomit. i would shake. my mother and the things around me looked 2-dimensional. all of the speech and other noises started to sound farther and farther away. i think this was fueled by fear/adrenaline?

I rarely look at people's eyes when they're talking because it makes me feel squeamish

I'd say it always causes a lot of discomfort and stress, psychological and physical (Tremors, stuttering, sometimes headaches)

It's painful - headaches, overload, etc

It's hard to describe, but it feels like I'm "overexposed" to their emotions and thoughts when my skin is already prickling because I have already figured out exactly what they're upset about and what they are going to say

Meeting someone's stare and trying to analyze whatever message they're sending to me is almost nauseating at times.

And I do get a tingly feeling in the back of my head and my ears raise.

I feel like there is something sort of collapses in my stomach before churning around in there. I easily start sweating

i have always had difficulties with eye contact. I always get a weird feeling in my stomach when i do it but i never knew what it was or where it came from

If I am forced to make eye-contact, my body becomes tense, my skin tingles, my jawline becomes somewhat numb

Eye contact also just makes my stomach twist and makes me feel like I want to vomit

It feels like something collapses in my stomach, and then it churns.

I start sweating.

***1.3 Pain***

Eye contact is physically painful

I get pain behind my eyes that fills my head and an uncomfortable/self-conscious (anxiety) feeling until I look away

It's like biting into a really sour lemon or licking the end of a battery. The feeling of tiny creepy crawlies shimmering under your skin making you cringe. Butterflies in your tummy trying to escape out through your head. Like the cold feeling when you realise your partner has been cheating on you

Same feeling when I accidently look at the Sun

It hurts like hell

As for what it's like, that's a bit tricky. Imagine that when you look someone in the eyes, you feel a fine rod of graphite extending from your eyes into their eyes. You can feel the graphite in your eyes extending out, and you can only imagine what the pain would be if you were to look away. The snapping of the graphite will surely bring you intense pain, you can't even blink. You have to wait for the other person to break eye contact. A few moments will last an eternity, whatever they say will be lost. Every ounce of concentration is on not breaking that fine rod of graphite extending from your eyes. Your eyes begin to water, you start to get antsy, your jaw clenches. It is an eternity of hell in just a few seconds.

It hurts; it's not unlike looking into the sun.

I fell physical pain in my eyes. Is the same pain and the same effect when you watch the sun in the clear sky directly. And it remain for at least 10 minutes

For me, it is painful.

I concur with the above posters who claim that making eye contact hurts and is unnatural

I don't do eye contact. It physically hurts, kind of like having a really bright light shone in my eyes

If I'm able to suppress all my instincts and do it anyway, my eyes start hurting within about three seconds

I find direct eye contact intense and somewhat painful

Looking into people's eye's, for me, is like staring directly at the sun.

When I was a kid, looking directly into a person's eyes, especially someone I didn't know, was like staring directly at the sun

Eyes hurt you, especially when looking someone in the eye, you need to blink to calm yourself.

It's a combination of an inability to concentrate while trying to look at someone and talk at the same time (I can't even look at the person in general, never mind their eyes, I have to look at something stationary when I talk) and a sort of instant of physical stabbing pain if I unexpectedly meet someone's eyes or feel like I have to look into them for some reason.

One of the reasons I couldn't look most people in the eye was that it was simply too painful

Honestly, it hurts. Every time I look into someone's eyes, it's like get a laser cutter to the brain and I freak out

My grandpa used to force me to look him in the eyes when he lectured me. It would feel painful, and I couldn't process anything he would say verbally or non-verbally. Eventually I felt numb, like my brain would shut down. I had teachers do this to me all the time as well. Rapid Desensitization/Flooding?

it was a very unpleasant feeling, a very disturbing feeling. And it really hurt,

they become almost painful to look at. Almost like looking at the sun. it’s just so very intense.

***1.4 Threat Response***

Also I get really defensive and impulsive when I try to make eye contact, almost like my brain perceives too much eye contact as a threat

I also tend to feel threatened by people who try to "force" eye contact. I had a manager like that once and I always felt like I needed to just go along with whatever he was saying to end the conversation as quickly as possible

if someone is talking to me and our eyes are looking at each other, then it feels very intense and direct and confrontational

Personally it makes me feel like it opens a channel and the other person can see into my private thoughts. I'm talking about a feeling that happens automatically, it's not something I think really happens. It makes me feel like prey that's been spotted by predator.

if I look at people too much I get caught up in a social dominance game whereby I try and win and I know it's not healthy

My problem is not so much making eye contact as receiving it. When you've met someone there comes a point when more than usual amount of eye contact is appropriate, say before kissing for the first time or when a guy tries to tell you they like you that way. For me that eye contact triggers a fight or flight response so strong that it overrides everything else, including my better judgement of the situation. I've never been one to back down, so for me it tends to be "fight". Most of my self control goes into not punching them outright and it feels like I don't have the braincells left to control the verbal & nonverbal aggressiveness. I do of course have enough presence of mind not to get physical, but the reaction really is that strong. I've certainly threatened some very nice guys with violence and scared some badly enough they were afraid to talk to me weeks afterwards. And these were men I wanted to see again.

I do get a moment of fear/panic before I need to look away.

That said, if the conversation becomes in some way confrontational and the other person attempts to bully me with a stare-down, I do usually avert the direct contact in order to prevent sensory overload. At such a point the conversation has probably devolved to where I am moments away from melting down into an angry emotional outburst. I aspire to excuse myself when I sense this coming on, though unfortunately too often I "freeze" then "fight" (verbally), even though "flight" would usually be the wiser choice.

glaring into our eyes isn’t a good idea either. This often has the effect of making us feeling challenged or threatened. Even if you don’t think you’re glaring, remember, we can’t read facial expressions very well and we can often think that you are

It seems too intimate or too aggressive.

I don't get any pleasure at all from eye contact with a lover. Just terror - and I get so instantly intimidated I'm almost angry

For me, eye contact triggers a fight or flight response

It feels threatening. I've only been able to look in the eyes of a small handful of people for more than a few seconds

I have to look away if someone on television looks straight into the camera.

It seems an act of aggression to me, like something animals do right before they attack and kill something.

I keep thinking my cat will attack me if she stares at me longer than five seconds. When she wants something she does. I'm never sure when a dog will jump up and bite me. Eye contact is threatening

All my life I've found prolonged eye contact confrontational, thus I believe I've never looked into anyone's eyes for more than a second at a time.

When I was little, we went to the Zoo. There was a panther in a cage. The cage looked too small, and it paced back and forth. It kept staring at me as it paced, like it wanted to gobble me up. When people make eye contact with me, it feels like it did when the panther stared at me, like a predator that wants to devour me: threatening, merciless, and cruel.

In addition it feels confrontational

I don't have that much trouble making eye contact, but I gotta say, people seem to have a way of making their "eye expressions" look dang scary for no reason.

Almost like a "fight or flight" reaction.

I think the most fear I have of looking at peoples' eyes is still with people I feel are threatening--that I'll see they think they're better then me, or will reject me--after all these decades I still cringe at that!

Also Eye contact is aggressive. There is a reason why people are advised not to look a grizzly bear, or rabid dog directly in the eyes. Eye contact makes most animals aggressive as all hell, and i don't think its any different with humans subconsciously.

It triggers fight or flight.

Because eyes look predatory to me, like some carnivorous animal's

**2) Invasion**

***2.1 Violation***

When I do it, it feels like they can look all the way into me and see EVERYTHING. So, I actually feel violated in a way

like being invaded by the other person, or like someone touching you in way too personal a way. It only lasts a second, as long as the glance, so it's easy to recover from, but it's-just-creepy.

And it's like I can can read someone's mind. Sometimes I think they are thinking awful things about me, other times I feel ok.

Sometimes when I have to look at someone it feels like I am touching them (even though I am not)

I was always confused by this because I am not inherently shy at all. I didn't feel it was shyness. It was an actual fear of the intimacy of eye contact. As if I was too naked

I have noticed that in my efforts to look at people's eyes more, it makes me perceive them more as objects and less as people. Looking at them is, more and more, a task that I do, and their face is an object that I have to look at to complete my task, so that for the time that I'm looking in their eyes they become a (distracting) object. It's odd that most people feel like it increases their sens of connection and personal contact, and for me it decreases it. It feels like I'm trying to do something

Either making eye contact causes me to feel like I'm invading their personal space and being intrusive and disrespectful (even though I know I'm not, I can't shake the feeling)

It feels like an invasion of personal space, and I can't shake the sense that the other person could be feeling that creepiness too.

Now post Dx I do not have to hide behind my persona. BUT because I know I possess the ability to act un-anxious if need be, I am able to relax into eye contact with people who interest me (be it sexually or philosophically ) and experience a deep emotional resonance with another person. (yes it can leave me exhausted after a while). It's like having the ability to not Stick out as anxious can offer me waterwings to float upon this sea of stimulation that is social interaction.

The eyes for me really are the windows of the soul. So when professionals seek to ignore the pain and suffering they can see manifested in the eyes of a person with Autism, what does it say of them that we should have to endure this discomfort for their sake. However well intentioned many professionals are their ignorance is still hurting us.

It also feels very invading -- it seems the same as if someone tried to have a conversation half an inch away from your face.

So I tried to consciously make REAL eye contact and came across two problems: 1. It felt really threatening and uncomfortable for me - I don't understand why. 2. I missed a lot more of what was being said because I wasn't able to focus on the words and the eyes at the same time.

The eyes are open doors. Liek houses with wide open doors pressed up against each other. It's like I see right into their private life, their hopes and fears, their most personal moments. I connect. Looking into eyes, kissing, sex, a lifetime together, staring for hours, one wants to lead to the other. Looking into eyes is like staring at someone naked and seeing them from childhood to old age in that state, and promising your whole life to them. I do want to do it (well with a few particular people!) but it feels so wrong.

I don't feel disorientated I feel exposed. I feel like the person I'm making eye contact with can see right through me and know things about me that I would rather keep secret. They say the eyes are the windows to the soul which is why I feel the need to avoid eye contact. I feel like I have more privacy if I don't make eye contact with others

Yes that's it, they see inside me and I feel exposed...

When I do have to look someone in the eye, it's like they're looking through me, like we can both see too much. It's an invasion of privacy

I feel like I'm invading the other person's space

When I accidentally make eye contact with strangers, I feel embarrassed and that I have to look away quickly because I've happened upon something that should remain private.

It seems too intimate or too aggressive.

Nervous like I'm being interrogated. I almost never do it.

I agree about feeling exposed. I also just kind of think it feels too personal

This. I feel as if they're piercing through me to the very depths of my soul, as if some piece of armour has been physically removed, leaving me extremely vulnerable.

If it is a guy, I feel like I'm flirting. If it's a gal, it still feels invasive.

I feel as if I'm spying on someone's inner thoughts, which to me is an inherently rude thing to do.

I can't keep eye contact, it's not just uncomfortable it feels like a violation.

The eyes are the gateways into the soul. Literally

I can't formulate thoughts properly while looking into eyes. It makes me flustered, as if I'm holding hands with them or something. It's embarrassingly personal

It feels a bit like "soul rape". Like someone is staring directly into my brain. Very unnatural and unpleasant.

I feel exposed

I don’t want to see their soul and I don't want them to see mine, it's like a depth that is unwelcome. It makes me feel really uncomfortable,

It's like I'm totally exposed when I my eyes make contact with someone else's

It's intrusive and invasive, like a stranger venturing into an intimate place where they don't belong

It's too intimate -- like they can see into my soul and real my innermost thoughts

Also, it makes me feel naked.

Eye contact for me is like looking at someones naked soul

It makes me feel naked

Also, I have never understood why people say that you can see someone's soul in their eyes, becuase i never can. I just see their eyes.

Soul rape is the perfect description.

it feels too private

When I accidentally make eye contact, I do feel violated

When two people establish eye contact, it generally means they have added another layer of communication that supersedes speaking and body language. You smile with the eyes. You also lie. It implies interest and understanding and comfort, or intimidation. For people who do not like such closeness, whether positive or negative, it's uncomfortable

As someone else put it, eye contact feels like looking deeper into each other's soul. For someone like me who is clueless to how others are feeling or what they are thinking, eye contact only betrays my confusion, makes me very selfconscious and that makes the interaction very awkward

To me, the eyes are the most personal feature a person can have, almost like the windows to the soul. Every time a person I don't know looks into my eyes or shakes my hand I feel like they are trespassing.

When I do it I do not feel I am looking into someones soul but it causes my eyes to become psychically uncomfortable

I feel very exposed, as if my soul was naked. It feels unnatural like a giant bore hole into my body. I feel judged on eye contact.

Why is it that NT's like looking into peoples eyes? Just seems so intrusive.

I don't know, it just feels like there's too much there. When I look into someone's eyes for more than a second or two its like I'm supposed to be seeing more than I am, like they're trying to see something in me that I'm not comfortable sharing. It's just feels wrong. It's a little better with someone I know and trust, but even then I generally look slightly aside.

I used to feel like someone was seeing right through me.

another thing is that when I was much younger or until even pretty recently it felt like if I was making eye contact that people could see right through me right through my soul and see everything there was to know about me.

I felt you know almost as if I was being raped on a spiritual level

I really felt like people could really just see me and, and touch me inside of my soul and do terrible things to that.

What I **feel** when I look directly at someone is that I am invading **you**, that’s **your** space. And to me eyes are very much windows to the soul

if you **look** at someone in the eye and you don’t know them it’s like you’re like it’s like you’re looking into their soul

***2.2 Fear of Conveying Private Information***

I always feel before I make eye contact that the other person and I will be able to see each other's thoughs, or something - something far more intimate than is reasonable in non-family members. Then my logic overrules the fear, I meet their eyes, and nothing happens: no telepathy, no drowning in the other person.

Then I talk so much with my eyes - people can read what I'm thinking and feeling, regardless of my body language and voice, just by looking in my eyes - that I don't like people knowing more about me than what I'm saying.

I've met people with "dead eyes". If I've met anyone who is as communicative with their eyes as I am, they certainly control it better. I can't control it

It's odd. I can stare at my dog's eyes forever. Then again, she probably has no clue what I am saying.

The other day one of the big bosses at work - a very quiet, gentle man - stopped to speak with me just to be friendly, and I thought I would die meeting his eyes. But I did it. Did fear show on my face? I have no idea. But I was feeling it big-time.

Personally it makes me feel like it opens a channel and the other person can see into my private thoughts. I'm talking about a feeling that happens automatically, it's not something I think really happens. It makes me feel like prey that's been spotted by predator.

i do not feel that i have a complete soul, and i do not want anyone to find out by looking in my eyes. my deepest personality is a supercilious facade, but i do enjoy being me. i just can not let people look through my eyes to see how shallow i really am.

I can also tell what people think of me, when I look them in the eye.

I'm bad at "Bluffing": When I'm tired, depressed, etc (Which is often; don't have a lot of money to treat my depression, so I'm sort of biding my time, possibly looking for a definitively part time job after a considerable length of unemployment. Probably couldn't handle much more than 20, maybe 25 hours.), I think that shows up in my eyes and is hard to hide…For as pleasant as I try to be, I know that to some extent, people can see through that and understand that I do have issues, whether it's my social awkwardness, depression, etc, and my eyes are the express route to that understanding, which I do find intimidating, being a person who is private with his feelings in the first place and who does fear some degree of judgment. I believe in the old saying about eyes being the windows to the soul for sure.

There are very few instances I share it and that's during interviews or business discussions, or spiritual topics. I feel sharing my gaze is sharing all of these things with others and I'm simply not ready to share.

When I have uncomfortable eye contact, I feel the other person can read my mind, or at least guess how I'm feeling. I think avoiding eye contact is an attempt at not letting the other person read your feelings. Even when you have nothing to hide, that feeling of mind-connection with the other person, caused by eye contact, is unbearably uncomfortable.

-I feel like the person can read my emotions -I feel like the person is judging me -I feel like the person is judging my looks

-I feel like the person can read my emotions

and a million questions whiz about through my mind... What do I do now? When do I look away? Is it going to get me into trouble if I avert my eyes? Am I in trouble? Why are they looking in my eyes? Do they think I'm lying and they are trying to "see" the truth? Are they trying to see if I'm not normal? Can they tell I am not normal? How can I get out of the room/area? When will they stop? Do I have to say something? Can I just look down at the ground? What about the wall? Can they tell what I'm thinking?

***2.3 Intimacy***

It's weirdly intimate, too intimate, and like I'm doing something I really shouldn't do. Even the shortest glance feels far too long and too "meaningful".

When I try to actually look at their eyes, I start to feel shy. It just feels so intimate, like I'm invading their privacy

I was always confused by this because I am not inherently shy at all. I didn't feel it was shyness. It was an actual fear of the intimacy of eye contact. As if I was too naked

Maintaining eye contact feels waaaayy too personal and intimate, and with someone I don't know very well that can feel downright creepy. It's almost like kissing someone and stroking their face with your eyes and who could possibly concentrate on what's being said in the middle of that?

I can look at people when they're talking to me, but it feels sort of weird. Like others have said, it's too personal, too intimate

if i find it too hard to give eye contact, it sometimes relates to not being comfortable making that connection with people. forcing me to look at someone is forcing an intimacy that does, indeed, have a tone of violation.

My brain interprets it as incredibly intimate, more so than sex even: it feels creepy that people would try to force that kind of connection before we know anything about each other

Eye contact is too intimate.

It seems too intimate or too aggressive.

It's too intimate -- like they can see into my soul and real my innermost thoughts

It is a form of intimacy that does not always seem appropriate

It's way too intimate, like staring straight into someone's soul and in turn having your innermost essence probed by them.

I am **in** your personal space and you’re in mine if you’re looking at mine. And suddenly people who are strangers are automatically to **me**, intimate. And it is not comfortable for me.

I'm fine with eye contact dealing with folks I'm familiar with and/or comfortable talking to

I can only look someone in the eyes if I trust them completely and if they already know me very well.

i hate making eye contact with people i don't know, and i can hardly do it with close friends

I can do eye contact when I'm comfortable with someone, I can almost forget about it - if I'm somewhere new then I don't find it comfortable so even with someone close to me I'll struggle

If I know and/or trust someone, then I don't get the same invasive/overwhelming feeling from looking them in the eye and I can easily do it, and feel how I imagine an NT feels doing it.

Unless it's my husband, and then I like the intensity, but with anyone else it's too much.

I do with people whom I know, like family, friends, co-workers, etc etc etc, or are willing to make some sort of interaction with, like cashiers, waiters/waitresses, train conductors, etc etc etc, or even people whom I hate or don't feel comfortable with. It comes naturally and doesn't make me feel a bit uncomfortable. But random strangers are different, and changes the way I feel about making eye contact, and it is rare that I give strangers a direct-eye gaze (although I can still)

it's easier with some people than with others. I don't know what makes the difference. As the OP suggested, it could have something to do with trust. Or it could be to do with the level of self-consciousness of the other person. When I make eye contact, if the other person and I both are selfconscious, we can both sense it, and it is very uncomfortable.

Actually making eye-contact: only to someone i love (happened only to one person so far), or someone i truly hate (a lot more often), to anyone else, it hurts like hell: even with my parents, eye-contact is painful

only look my sister at the eyes, and I do look at other people when talking only if the topic of conversation fascinates me, and I feel I have something important to say (and after I say it, I suspend eye contact).

I also find eye contact easier with people who wear glasses for some reason, maybe because I can focus on the glass or frames instead ... or simply because of the extra barrier, who knows.

When you've met someone there comes a point when more than usual amount of eye contact is appropriate, say before kissing for the first time or when a guy tries to tell you they like you that way.

Eye contact is in inherently uncomfortable thing for me, that I can only achieve with those whom I have a degree of intimacy or trust with.

for me, it's not that looking into someone's eyes is painful, or disturbing. I can do it with my wife and children easily. I relax into them. For others, even my brother or mother, I can't do it for long. It's just that I am *most comfortable* fixating on something else other than their face or eyes while I am talking to them.

I usually only make eye contact with my close people, like my son, partner, parents.

If I don't like someone I will not look at them at all. I look at the ground or to the side

I only make eye contact with people I know very well.

I find eye contact easier with members of the opposite sex. I find all social things easier with members of the opposite sex.

I enjoy looking to eyes if it's some cute girl and really pleasant to look at

I don't mind eye contact if I'm familiar with that person, though.

If I get to know someone really well I can make eye contact here and there without getting uncomfortable but otherwise its nearly impossible.

I didn't have any trouble giving eye contact to people whom I felt comfortable with. It's the people whom I was scared of, that I had a great deal of making eye contact with. The more mean spirited the person was, the less likely I was to look them in the eye. I'm still like that.

Eye contact for me is rather simple. I have it with people I like, and find interesting, and especially if I have a crush on somebody.

I do better when it's one person, but in a group, the more people I'm dealing with the harder and harder it is to make eye contact with any of them.

I've noticed that with people I'm comfortable with, such as my mom, it's easier to look at their eyes a little.

Eye contact comes naturally when I'm talking to anyone, whether it's someone I know or just the cashier at the check-out in a supermarket. But eye contact with strangers who I needn't talk to is like one of the most hardest things to do

It's worse the more someone is unknown to you. It's never entirely comfortable though even when you know the person well.

If I only glance from time to time I can get through it a lot easier. If It's someone I'm very close to -- a family member or a partner -- it's much easier. If it's a man to whom I'm attracted but haven't revealed my feelings to, the anxiety increases about fitfy fold.

It is a form of intimacy that does not always seem appropriate

The only person I've made eye contact to on a daily basis is my girlfriend

I can easily make eye contact with people I can fully trust.

It just doesn't make sense. Why would you want someone to look into your eyes? Unless maybe your in love with them but otherwise...

i can look at animals looking at me. i do not like to look at humans looking at me because they have millions of secret things going through their minds.

In fact, there are some people, like my son, that I have no problem looking into his eyes...but others, like my husband, when I do I just see evil. I think for me, the eyes say a lot and I don't like what I see in most people's eyes.

I can only look into the eyes of my wife and children in a normal and natural way. That feels great. I generally cannot maintain eye contact even if I try out of social convention. It's like all eyes are magnets and other peoples eyes repel mine. During conversation I get a feeling of panic about whether I should try harder and what is expected of me.

But it does depend on the person and the situation. I don't have a problem with maintaining eye contact with close family and very few friends. With others though, there's a conscious awareness of what I'm doing with my eyes and it's quite distracting, especially when I'm doing the talking. Speaking in front of a group of people is the worst, because I have to deal with multiple sets of eyes that are glued to me! When I'm listening it's not so bad, but it's easier to focus on their words when I'm looking elsewhere. There's no 'magnet effect' when I'm looking at eyes that aren't gazing into mine.

I don't know, it just feels like there's too much there. When I look into someone's eyes for more than a second or two its like I'm supposed to be seeing more than I am, like they're trying to see something in me that I'm not comfortable sharing. It's just feels wrong. It's a little better with someone I know and trust, but even then I generally look slightly aside.

I even have trouble looking myself in the eyes in the mirror.

I can make eye contact with certain people. I can look at my siblings and make complete eye contact with them (no laser pain) but I generally have no clue what's on their minds. Contrasting this, I can make eye contact with other autistics and generally recognize if they are autistic (it's weird but I'll bet I'll be wrong some day. I can also do it with siblings of autistics

I actually really enjoyed eye contact when I was in a relationship before, but only once I felt comfortable with her.

If I feel anxious about a person I tend to avoid eye contact completely. If I'm very angry with someone, I tend to give them a lot of eye contact or none at all depending on if they are in my way somehow or if I can go another direction.

Eye contact for me is as natural as breathing, but when it comes to passing strangers, I just can't do it. It becomes as scary as entering a lion's den.

It's weird how something I find so natural most of the time becomes extremely unnatural in certain situations.

It's mainly for people who I don't know. People who I know and are comfortable with, it's much easier.

If someone I know, and comfortable with becomes angry or disappointed with something I've done, then it once again is uncomfortable to look them in the eye. Maybe that has more to do with a sense of shame in those instances.

'Cause when I do I'd often freeze into their eyes, I wonder how I look. However, with someone who doesn't seem to have a darting intimidating look like with a friend of mine who has crossed eyes, I can freely make eye contact without engulfing into its sight.

the only time extended eye contact is appropriate is if it **is** an intimate relationship so again, uhhh between me and my grandchildren between me and my children, um you know that this is going to be fine, between me and a close lady friend or something, it’s going to be fine uh for a limited period of time. The only time it’s intense and extended is during sex.

Eye contact is easy for me IF I trust the person I'm talking with. BUT if I don't trust someone to care about me, I freeze if they make eye contact with me...it feels as if they can look inside me and 'see' the real me. The one I keep private and allow out only with certain people.

**3) Sensory Overload**

***1.2 Audiovisual Integration***

I can force myself, but then I can't think straight. I can either articulate a thought, or I can look you in the eye

Its like everything goes distorted and I can't make sense of things, I become disorientated when I do it. It

And I couldn't listen to him and make consistent eye contact without tuning out what he was saying.

If I'm talking, I can't make eye contact until I get my thought out.

I'm not able to talk fluently while making eye contact and I go into stupid mode where I can't naturally make a sentence flow together.

On a more serious note, eye contact interrupts the flow of my thoughts. It is as if the eye contact itself becomes the primary thing of which I am cognizant and thinking takes a back seat. Depending on the amount of mental effort required to maintain the conversation, eye contact can be very disruptive. You can have eye contact with me or you can have conversation.

I don't feel any anxiety or anything. It's just distracting and makes it difficult to make sense of what the person is saying.

I stop hearing what is being said to me....

I can look someone in the eyes but I will be very distracted if I have to pay attention to that instead of what they're saying. It's much worse if I'm trying to talk while making eye contact versus listening.

It's a distraction and gets in the way of talking.

When I was younger, I'd dig deep into my head when talking to someone, and as a result, I wouldn't focus my eyes on anything in particular. I found looking into people's eyes to be distracting and intimidating.

when I do make eye contact (even now, having trained myself to tolerate it somewhat), that tends to overwhelm my ability to process anything else.

I can't concentrate while making eye-contact, particularly if I need to listen to what the other person is saying to me. It's like I need to shut off the visual input in order to completely process the aural input. It's ok if the visual input is something neutral, like staring at the ground, but looking at a person's face is just too much visual information.

Yep, me too. I find that the moment I look them in the eyes the voice in my head starts talking, which means it's overwriting what the other person is saying and the images their words conjure in my head (if I'm trying to listen to them) or it's overwriting what I was thinking and picturing and trying to express (if I'm trying to talk).

I usually try to look at people's eyes, especially when talking to my children's teachers. However the moment I see their eyes I think "Ooh, pretty blue color", "the mascara's smudging", "Very dark, I wonder if she's mixed"...etc. Then my eyes start to dart around and notice their hair, earrings, necklace, glasses, cleavage...etc. and miss most stuff they're telling me. However if I don't look at them I pretty much get everything they're saying. I wonder if I should bother to look at them at all. But I'm afraid they'd think I don't care about what they're saying.

I'm busy thinking about looking at their eyes more than listening to what they say, I don't know how people do both!

The irony is frustrating - don't make eye contact, and I'm able to concentrate on the conversation, but people think I'm not. Make eye contact, and I'm unable to concentrate on the conversation, but people think I'm more attentive.

Whenever I find myself looking at someone's eyes, thoughts like "Hmm, that's an interesting texture." and "So what's so special about eye-contact?" float across my mind, and I tend to lose my focus on the matter at hand.

Maintaining eye contact feels waaaayy too personal and intimate, and with someone I don't know very well that can feel downright creepy. It's almost like kissing someone and stroking their face with your eyes and who could possibly concentrate on what's being said in the middle of that?

The only problem I have with eye contact is that I need to be able to look around in order to talk. If someone asks me to describe something that happened, for instance, I'm not able to access that information and translate it into works if I'm focusing on someone's eyes.

Yes, I can't look at someone and think about what I'm saying at the same time. I've tried to practice glancing at them now and then, but I guess I don't do it subtly enough, because they look sort of startled when I do that, like I'm a detective who's eyeballing them to get them to spill the truth...

I have had people look where I am looking, or move sideways in to my field of view. It is just how my thought process works - I have to look away so I can speak what I am thinking.

I've observed in movies etc how people look at one another (not specifically watched movies with this intent, it's just something I've observed) and seen how they change which eye they look at. So I do try to change which eye I'm looking at, however the downside of this is that I might miss what they're telling me as I get so hung up in "changing eyes".

I can't stand looking in someone's eyes. It doesn't feel right and they are too distracting. If I make eye contact, I can't process what you're saying.

I just can't talk to the person and look at them at the same time, so I'm usually looking elsewhere.

I can't look at someone and speak at the same time. It's either one or the other, and usually I take talking over eye contact, because it's more fun, generally speaking.

If I try hard to do the eye contact thing then I have little if any recall of what was actually said to me. If I am looking at them I am more likely to say weird things that make no sense or will start stuttering struggling to find the right words. Its so weird how that happens. I mean it makes no sense to me why I can't function if I look at someone but its like the brain short circuits.

When I speak I usually look down and to the side, if my body is facing someone, and similarly when listening. Otherwise I can't really hear them.

If someone is talking to me and I make eye contact with them, it is usually harder for me to follow what they are saying. I usually start tuning out what they're saying automatically.

Whenever I have a conversation with someone who is very insistent about making eye contact, I have the choice to either look at them, which is extremely uncomfortable and will result in my not understanding anything that they are saying, or to look away and just nod or something to indicate that I understand. I think the latter is better, because it's better to really understand the person and be able to respond than to just look like you understand. So for me it seems to be more of an information processing issue than feeling that the eye contact is aggressive, though I do find it uncomfortable.

I have a very difficult time with eye contact while speaking if I'm also trying to formulate my thoughts. I don't know why, but my brain just doesn't work if I'm looking at someone. All while growing up, my mom told me that I needed to make eye contact, not in a forceful way, but in a "this will help you socially" way. Unfortunately, time hasn't changed anything. If I'm trying to think of what to say, especially if I'm explaining something, and I force myself to make eye contact, I often forget what I was going to say and then panic because I don't have anything intelligible to say. I tend to look at people when they are talking and then look down when I start talking.

The feelings eye contact causes seems to be a little different for everyone. For me, it's distracting because now I am thinking about their eyes and their face, and not their words.

I often think and listen better when I'm not having to think about continually being concerned what proper eye contact the person wants or needs.

There is so much information coming in from a speaker that I try to assess as the communication is happening. If I have to look in the eyes, I lose everything else, especially what is being said.

I find if I look at the face, I loose track of what they're saying.

I kinda fail at eye contact, makes me severely uncomfortable and hard to focus.

If I am looking at someone's eyes there is an information overload and I'm unable to concentrate on understanding what they are saying or formulating a response. Looking at other parts of their face isn't something that works very well for me either - it's not as bad but still distracting.

Its hard to explain how the discomfort of looking people in the eyes feels but its very unpleasent and prevents me from paying attention to them.

For me, eye contact contains too much information, that I am not able to properly process.

I get lost when I look into a person's eyes. I stop listening and just...stare. Feel their eyes locked with mine...look at the pattern in the irises. The words stop coming in and I either become absorbed in the look of their eyes or I feel creepy all over.

And while I'm analyzing her eyes and face, I realize that she's been talking and I haven't heard a word she's said. And there she is, waiting for my response.

So I tried to consciously make REAL eye contact and came across two problems: 1. It felt really threatening and uncomfortable for me - I don't understand why. 2. I missed a lot more of what was being said because I wasn't able to focus on the words and the eyes at the same time.

Single-channel attention: it's hard to hear what people say and look at them at the same time.

I can't focus my attention on what the person in front of me says while making eye contact

It is too overwhelming. I see thousands of pictures when I look at a persons face. I cannot process that data and the voice.

It is more information than I want to deal with and it scares me.

When I make eye contact I forget what I'm trying to say or achieve. It feels like when I look into a person's eyes that I'm seeing almost too much to deal with.

It means for me that a good portion of my concentration on the conversation is now refocused on maintaining eye contact, along with the distracting thoughts of where my eyes should be which of course means that I'll lose much of what is being said to me in the process.

other than that, i do not need to see what people's eyes look like and so i never look at them. their voice is what i listen to if i remain in their social vicinity, and my visual tracking system powers down automatically when i switch to audio mode.

As it is difficult to anticipate what will be communicated in a conversation or where it will go. I compensate for this, by focusing on a limited set of information being communicated (i.e. what is being said). As the other information coming in (say, from facial expressions, eye contact, body language) is too overwhelming to process quickly.

I very much have the listening/looking problem. I just suck at multitasking period, but that in particular. I can't talk while doing anything, even if it's autonomous. Didn't even realize this was a thing that was common in autism.

It distracts me from what me and the person are trying to talk about because I have to consciously try to make eye contact, or to make it 'correctly'.

It feels really awkward and uncomfortable. I don't see why I need too look at people in the eye especially because if I do all I can think about is looking away which distracts me from what they are actually saying.

-It distracts me from the conversation...I cannot really listen and make eye contact, I seem to perceive that as two totally different tasks so I can really only focus on one or the other.

If I don't focus on the eyes I can actually listen and not just hear the noise.

I get uncomfortable with eye contact, like many other people here, I also cannot talk to someone if I'm looking directly at them, it confuses me and I lose track of what we're talking about. If I want to be able to concentrate on our conversation, I'll have to be looking at the ground or at something inanimate that isn't sending me emotional signals that I have to decipher and understand.

and if I have to talk it's almost impossible to think about what I need to say because the sensation is too distracting.

I am a hopeless multitasker, and for me, making eye contact plus conversing is multitasking.

It just freaks me out. Can't think straight if forced to do that.

It also shuts down my ability to think. I used to think that that was just due to anxiety, but I'm on a new med that reduces my anxiety a lot and find that eye contact still locks up my ability to think.

And again, like everyone else -- when I look directly into someone's eyes, I completely forget where I am in my conversation. I start stammering and forgetting words.

It's a combination of an inability to concentrate while trying to look at someone and talk at the same time (I can't even look at the person in general, never mind their eyes, I have to look at something stationary when I talk) and a sort of instant of physical stabbing pain if I unexpectedly meet someone's eyes or feel like I have to look into them for some reason.

it's difficult enough to process what someone is saying and then be able to translate my thoughts into words. i don't need added stresses and distractions.

I rarely have the (natural) urge to make eye contact, the vast majority of the time it is a (nurtured) response. I'm actually quite good at multi-tasking but making and keeping eye contact, literally, seems to take all of my focus and leaves me basically incoherent. It simply causes me distress.

But when I am expected to make eye contact with the manager I feel very uncomfortable, and it is hard to listen and look at the same time.

So when I stare I end up uncomfortable and I end up having to spend more effort looking at them than I do thinking!

But looking into the eyes (when in close proximity) is very uncomfortable. I can do it (if I think about it). But then I cannot focus on interaction.

Since I already have difficulty processing information in a verbal format, making eye contact with a person while the person is speaking means I likely won't be able to understand what he/she is saying.

I can't seem to keep track of what people are saying, I can listen better if I'm looking away from them, if I make eye contact my mind just wanders off its like a go into some sort of scary trance

My grandpa used to force me to look him in the eyes when he lectured me. It would feel painful, and I couldn't process anything he would say verbally or non-verbally. Eventually I felt numb, like my brain would shut down. I had teachers do this to me all the time as well. Rapid Desensitization/Flooding?

Think of all the information we have to take in about that because every single face is different, eye color, hair color, the shape of a person’s eyes, nose, mouth, any wrinkles or spots they have, the way their face moves as they talk, and their expression changes, or how their faces moves as they breathe, how their eyes move, blinking, eye lashes, eyebrows, possibly facial hair or scars. This list is getting long so I’ll stop here, but that’s not all of it, nowhere near. Looking at people’s faces can be overwhelming for us and that’s why we don’t like doing it.

one of the reasons I have, it’s hard for me is that when there’s just so much information in people’s eyes and there’s so much information about the way they feel, what they’re thinking, the way they feel about you, and it becomes really intimidating and overwhelming just because there’s so much.

It’s very difficult to look at somebody’s face and listen to what they’re saying at the same time.

I can get very overwhelmed just looking at someone’s eyes

I just feel that I can’t think of what I have to say until I take my eyes off of the camera or off of the person’s eyes or whatever and people might think I’m being rude but really I’m not I'm just trying to think of everything I'm trying to say because there’s so many things in my head that I'm trying to filter out

so sometimes okay uh I feel like I can be lost in my own thoughts and that can like cause people to think that I'm also being rude or not listening to them but I, I get caught in my own thoughts um to often like even right now it’s hard for me to dissociate er not dissociate um... I guess you can say disassociate myself from one thing from one thing in my mind because there’s so much going on

I'm pretty good with it but see you can see how there’s like a space and then like I stop I pause I think ‘cause there’s **so much** like it’s it’s like a big pile of not crap but like big pile of **stuff** and you have to search through every single thing and you have to first you have to put everything in order and **then** you have to search.

if I'm in conversation with somebody and I need to listen to what they’re saying then I can’t make eye contact because if I make eye contact I can’t listen to what they’re saying

it’s almost like I can’t **hear** what they’re saying at all like a bit like going tone deaf as soon as I make eye contact because it just doesn’t go in.

then to try and make eye contact and me talk that's extremely hard yeah really not easy at all

I tend to look at the things that are less stimulating the face is a very stimulating thing to look at its there’s so much going on. There's so much going on around in the world that I often look at the floor or I look at something fairly [pause] bland like um like a blank wall or something. Because that makes it easier to concentrate um ‘cause I'm not I'm just basically not very good at multitasking.

When you look at a person’s face, there’s so many details in a person’s face, so many things going on at the same time, so many tiny details of information about emotions, and about feelings, and expressions.

***3.1 Energy Exertion***

Eye contact is so "exhausting" that it makes me forget everything else, and I can't follow or memorize conversations properly anymore.

When I am engaged in serious conversation with most people, I find it hard to look in the eyes at the same time. I force myself to do it, but it is very tiring and I have to struggle even more to work out what they are saying

It requires huge amounts of brain power to initiate and sustain, this is to say if I am making eye contact it is the only thing on which I can concentrate at that moment.

But I'm basically there, treading water, every conversation: I'm unfocusing my eyes while looking at their nose and filling in the blanks from their end of the conversation from what I don't get from voice tone and word choice based on my ToM skills. They have no idea how hard I work. No wonder I tense up when people come to talk to me and shake after they leave, even if it was a pleasant conversation.

It sips my energy into my core like being hypnotized by a cold-blooded energy sucking vampire.

So, for me it feels like I’m using up a lot of energy. The longest I can stare at someone in the eye is from less than 2 to 6 seconds at the most. Then it gets tiring.

It often feels like, like I’m being blocked mentally (puts hand to head) from giving eye contact, something stops me, something is forcing me not to make eye contact.

yes it does take a lot of force, a lot of energy for us

One of the reasons is that it really uses a lot of energy to make eye contact.

It’s like very draining as if you’ve been working very long period of time. So it’s a lot of work to make eye contact. Especially when I really start to do it you know it really uses a lot of my energy.

It just feels like..an example if I had to higher my hand like this *raises hand above head* and yeah it feels like it’s OK like after 2 seconds but if you do it for like 10 minutes or like even 5 minutes then you can can pretty tired.

People with aspergers their eyes get tired because feels like a chore and it’s not naturally inside of us to do that.

I have to um reallyreallyreally try hard to concentrate.

**4) Social Nuances**

***4.1 Feels Unnatural***

Eye contact for me though is an extremely uncomfortable thing that doesn't come naturally in any way

Direct eye contact seems unnatural and awkward to me

I hate it. It just seems wrong and unnatural

Eye contact has never been natural for me

I concur with the above posters who claim that making eye contact hurts and is unnatural

I do have a problem with eye contact - it feels unnatural and uncomfortable but I force myself to do it because I know I should

Eye contact doesn't come naturally to me, and I can't really read eyes anyway. I usually focus automatically on the mouth, even in videos and still images.

First of all, it's just not a thing that comes naturally. I never had the urge to look into anyone's eyes, it's just not a thing that I do.

To me it feels extremely awkward and unnatural, almost to what an NT female would think of someone staring at their breasts. As well, my mind usually goes completely blank and I can get overwhelmed with emotions when I look people in the eye if I don't know them.

It does not come naturally to me, and I had to learn to do it. Since it's not natural, it can actually distract me from whatever the topic of discussion is, in order that I pay attention to my eye contact with the other person or people with whom I'm talking.

Largely because it does not feel natural to me.

For me it just feels extremely unnatural. As soon as I look into someone's eyes my mind just goes completely blank. I'm no longer listening to what they're saying and all I'm thinking about is the facial expressions I'm making to try and look like I'm a normal person who's actually engaged in what they're talking about

it's awkward and it doesn't feel natural

It doesn't come naturally

Now it just feels really unnatural

I rarely have the (natural) urge to make eye contact, the vast majority of the time it is a (nurtured) response. I'm actually quite good at multi-tasking but making and keeping eye contact, literally, seems to take all of my focus and leaves me basically incoherent. It simply causes me distress.

It doesn't come naturally to me.

It makes me very awkward and uncomfortable. Even with my husband, I often talk to him without actually looking at him

It’s just not in our wiring to stare people in the eyes.

People with aspergers their eyes get tired because feels like a chore and it’s not naturally inside of us to do that.

But I do believe it’s a learned behavior from social cultures and I don’t believe it comes naturally.

I can do it and it’s not I'm not saying that it’s [pause] something that I'm not able to do it’s just something that’s pretty hard to constantly do over and over when it’s not comfortable when it’s not something that comes natural

***4.2 Confusion About Appropriate Use of Eye Contact***

Normal is letting one's gaze wander from one eye to the other to the nose and back

My big problem for a very long time, and probably still, is to determine how much eye contact is appropriate.

I can really not figure out how long you're supposed to maintain eye contact, I know I don't make enough eye contact as my eyes dart around everywhere to avoid looking at the other person, but when I have to look them in the eye it's impossible to figure out when to break eye contact

It baffles me that the smallest millisecond too long is the difference between being normal and being slightly 'off', and that people make decisions about you based on first impressions

I've actually been noted for an unrelenting gaze, which I realize is very unusual for a diagnosed Aspie.

I am 39 and I still have trouble with it and I don't know how much eye contact is appropriate I either don't look at the person or I stare

i'm not a look-away-er ( as you put it ), i'm a starer and always have been...and NT's have been dissing on me for as long as i can remember about it. dont make anyone uncomfortable if you can help it; but dont allow them to make YOU feel like your in the wrong either ! !! !

I'm too busy measuring how long to maintain contact

I'm still trying to figure out how best to do the "check-in", where you re-establish eye contact every so often to show that you're following along. I glance at them briefly in the normal way, but my eyes don't quite manage to meet theirs. It must look pretty weird.

And generally when I do force myself to make eye contact I do so with such direct laser beam intensity that I either scare/creep the living hell out of the other person. Can't win for losing.

I try to avoid eye contact with most people, and when I have to use it to be polite, it's forced, and most people say it comes across as "faked"

If you make eye contact for more than four seconds (maybe less, but at least four) you're staring at them and they get uncomfortable. Also, remeber to blink

I also INEVITABLY stare at girls when they attract me. And I love when they reciprocate it. I know they have no idea why I stare at them. But holy s**t, I can't avoid it, for some reason.

However the other problem I created by trying to "correct" the looking at people was sort of a fixed gaze that I would turn on people in an attempt to show them I was paying attention when they were talking (I couldn't really pay as much attention but I could maintain it). More so if it was somebody I was closed to and cared about, and didn't mind studying their face/eyes, or I could just sort of give them an unfocused stare. Ironically, this staring actually made THEM uncomfortable. So it is a mistake to tell kids that eye contact is the key. In fact forced eye contact also seems to make other people uncomfortable (though I think they feel like I'm listening more so than staring at the floor/wall nearby, but they don't like it). I only remember liking to stare into the eyes of people I was interested in romantically. However, this made them uncomfortable and I did get asked to stop.

TBH, I personally don't have a problem with eye contact unless it's towards people I dislike. Looking in their eyes is like looking at a monster! My eye contact is actually on the other end of the spectrum in that I stare

Apparently, the normal amount of eye contact to make is about half time time during a regular conversation, or twothirds or three-quarters of the time when conversing with someone close to you or having a more personal or intimate conversation. I found this statistic alarming because I knew I didn't have a hope of doing anything like this

There are actually key moments in a conversation where people check to see if you are making eye contact with them, as for the rest of the time you want to keep eye contact anywhere from 30-40% of the time ideally. You can get by with less than 30% but if you don't reciprocate in those key moments than they will think you are being distant or assume something else. It's hard to define what "key moments" really are, but if I were to take a stab at it I'd say it's when there is a bit of important information or novel idea, a pause, a laugh, a facial expression towards one extreme of the plethora like a smile or smirk as opposed to a slightly "happy looking countenance"?

Namely, when I look at others, they get creeped out *amazingly* quickly (2 seconds or less seems typical). I'm not aware of, I dunno, deliberately making googly-eyes at

somebody, but that tends to be the sort of reaction I get. I often only notice I've done this as I notice someone startle, glare back or fidget until I look away just a little. Eye contact has never been natural for me, so when I followed that rule, I unnerved my classmates. I stared. They didn't like it. And it did not really help me be a more effective listener.

people find my eye contact uncomfortable and uneasy, many people, including my mom, have said it feels like I'm staring right through them or that I'm like a robot. I actually destroyed chances at friendship because of my unpleasant stare.

I've been practicing for most of my life, though, so occasionally I have natural eye contact. If I'm saying something important or need reassurance, I look someone in the eye. But those moments are rare. Does therapy train you for eye contact? Because I'd like to learn how not to seem creepy.

This is going to sound odd, but I didn't realise I didn't make eye contact until about a year ago. I have usually looked at people's mouths when they were talking and I assumed that was "eye contact" because I was looking at their faces. If not looking at their mouths, I wasn't looking at then at all…But suddenly I had the realisation that actually most people look at other people's eyes - and that is what everyone refers to as eye contact. What a revelation!

Either I stare too intensely or I don't look at them at all, Or, I look at them inapropriatley

think it's interval-based - something like one-half to three seconds on, three to seven off. At least, that tends to get me by in most social situations, and it gives me a little time to breathe after making contact.

If I am with anyone other than close family I feel that I have to keep the eye contact going - I just can't seem to glance away then back again. I don't know how long to look away for or where to look - that is what you're supposed to do isn't it

If someone is talking I can look at them, although probably my gaze is more unflinching than perhaps it ought to be

When I was a little kid (and I'm a very visual person) I used to get smacked for "staring." I got smacked for "staring" quite a lot, too. I don't think Mom realized that I wasn't intentionally staring at people and (in her perception) being rude, I was simply trying to read their expressions (which I am very poor at doing.)

I don't even know if I properly use eye contact, but I certainly don't look into people's eyes when it's discomforting.

I never use eye contact outside of conversation. Is eye contact used outside of conversation? If so, how?

In which cases is eye contact used without talking? And is it appropriate to use it without talking at all? Please state its most common uses without talking.

I don't know what normal eye contact is or when one should look away and I feel it is easier to either not look at all or to stare in to their eyes permanently. So which is better, especially for a date; stare or don't look?

I always think people are staring to judge, which I always thought was wrong, especially if you're not doing anything to attract such stares, which I know I don't.

But I suppose it's another double standard. It's ''OK'' if NTs do it but an Aspie is always told it's socially unacceptable to do is and so ''mustn't'' do it.

When I was younger, I was told that I tend to make too much eye contact. "Boring into and ripping out my soul" is how one person described it. Up until then, I always thought I had "good" eye contact LOL! Since then, eye contact makes me feel self conscious because I am never sure if I am doing it "right

I dont try to make people uncomfortable with eye contact but when I force myself to make eye contact I inadvertently cause them to be uncomfortable. I just dont understand what is too much and what is enough.

I don't ever know what's appropriate in terms of how long to maintain it, when to look away, and what to do with my eyebrows/expression while doing it.

I never knew the "look someone in the eye" thing was a real thing that people do until I was 23 and someone asked me what color my girlfriend's eyes were and I had no idea.

i have a tendency to stare at people, so too much eye contact.

It's too confusing. I don't know when I am supposed to look at the person, for how long, how long to look away, how to communicate that I am listening and not appear lost, communicate interest, "smile with my eyes", etc. Too many rules!

I feel like I'm staring at the person and that my eye contact etiquette is all wrong and I'm making them uncomfortable

The main reason I don't like to make eye contact is because I used to stare at other people when I was younger and they would say, "What? You've got an eye problem?" and then I would say "No.", and then they would say, "Well then don't look at me!" It was mostly kids who said these things to me, but I'm still uncomfortable with making eye contact to this day

If I force eye contact, then it is not spontaneous and it's so intense that I'm making the other person very uncomfortable and making him/her look away

I have the reverse kind of issue where I sometimes make too much eye-contact so I try to avoid it. I had the thing where I looked at someones mouth once because I didn't want to make too much eye contact. I get the feeling that may be as "bad" as too much eye contact/staring

First I'm not looking at you enough, now I'm staring?! What's the difference between looking and staring? Do you expect me to break eye contact, look at some other random thing, and then reestablish eye contact every five seconds or so? Great, now I have to remember to do that too. What should I glance at? The clock? No, that's rude. The light? No, it's fluorescent. It's bothering me. The wall! Okay, I'll look at you while randomly glancing at the wall.

On the times I do make eye contact I feel like I must have an insane stare. Some one further back said something about it feeling like a bomb about to go off. Nailed it.

I find it uncomfortable to maintain for long, and am never quite sure how much eye contact the other person expects

Why is it that NT's like looking into peoples eyes? Just seems so intrusive.

Much like others have said, I don't do much eye contact because it doesn't come naturally to me. I don't really know the appropriate timing, and I feel at risk of staring for too long, which could be taken as a threat-stare or a sexual stare.

I'm always afraid that I'll give eye contact incorrectly. Sometimes women smile at me and give eye contact and I don't know how to reciprocate other than by smiling awkwardly and looking away from them

I have this irrational fear that making and maintaining eye contact with another will be misinterpreted as staring

I thought I was making eye contact. I could see her eyes, if I could see her eyes in my opinion I thought was making eye contact, at least back then it was you know until pretty recently. And so I could see her eyes but you know I wasn’t looking directly into her eyes, and I didn’t know exactly what she meant, and she kept on smacking me.

another thing you want to work on is observation. You want to notice what other people do what people who umm seem to have not make other people uncomfortable with their eye contact do and then you can try to mimic that.

I often find that people [pause] seem to **like** you to make eye contact which is kind of another bizarre thing.

***4.3 Self-Consciousness & Embarrassment***

The internal monologue starts up about whether or not I should keep looking into someone's eyes, or if I should look away cause I'm creeping them out, or whether I need to look at them because they now think I'm not paying attention...(which I'm not, because I'm too busy thinking about eye contact).

The conversation quickly falls apart as I start thinking more about looking/not looking instead of talking. I concentrate too much on the mechanics of it - I should maybe look away now or concentrate on the forehead instead - oh, I just did that so I'll look at the left eye now. Hang on, they've looked away? Did I look for too long? And so on... Er, sorry - what was that you were saying

I can make myself do it right, but it never stops involving conscious thought to keep track of how long I've been looking/not looking. It's very hard to do that and think about what you are saying/doing in a conversation.

I think when I'm self-conscious and rather force myself to have eye contact, it often makes other people uncomfortable and they look away

it's easier with some people than with others. I don't know what makes the difference. As the OP suggested, it could have something to do with trust. Or it could be to do with the level of self-consciousness of the other person. When I make eye contact, if the other person and I both are selfconscious, we can both sense it, and it is very uncomfortable.

If I force myself to make eye contact I can't hear the other person as my mind is filled with...I'm feeling really awkward, I don't like this, how long should I look for, it feels though I'm staring, this is painful, do they notice how awkward I am,

On the first second we look at the person normally, Then we have a sudden self-awareness that triggers a flow of questions that comes stronger and stronger, "Oh I am looking at him"

"Does he looks at me the same way I am?" We start to loose track of the conversation "I should look him into the right eye"

"Or maybe the left is better" We start to feel insecure "What was he saying"

"Dang his eyes are moving too"

"He sees my discomfort" We feel the pressure raising as the questions come faster and faster, less and less focused on the situation.

"I remember talking to him last year" And then the need to discharge, just looking elsewhere resets the cycle.

sseeing this flickering look of wonder in their eyes, and i know there must be a lot going on in their head, a lot more than what is showing, and i basically go into paranoid mode. ( they can SEE i'm not listening oh god what can i answer * "mh mh?"* little nod* oh my god did i just nod about her cat dying jesus i need to get out of there) that's basically it. and that's when i start off relaxed lol.

I often think and listen better when I'm not having to think about continually being concerned what proper eye contact the person wants or needs.

But when just passing a stranger, it find it so difficult to make eye contact for some reason. It just feels awkward and unnatural, and feels like a really big effort to do so. I then get all these self-conscious thoughts, like if I don't make eye contact with a stranger they might think ''what's wrong with that girl? Or what's wrong with me? Most people naturally look at each other when passing, so why hasn't she?'' And then if I do force myself to make eye contact, I keep worrying that they might think, ''what's she looking at? Do I look funny or something? She didn't have to look at me, I didn't know her!'' I know these are mostly irrational thoughts, but I still can't help believing them. I just want to go back to the days where meeting a stranger's eye was subconscious.

I now know one of the important things is not to force yourself to have eye contact and not to think about eye contact. If you think about eye contact, that makes you very self-conscious and awkward, which makes the other person very uncomfortable as well. I realize good eye contact happens rather naturally without being too conscious of it.

It means for me that a good portion of my concentration on the conversation is now refocused on maintaining eye contact, along with the distracting thoughts of where my eyes should be which of course means that I'll lose much of what is being said to me in the process

I've recently, actively caught myself not making the kind of eye contact I had previously learned is the amount and manner that the rest of the world mostly makes. I have some self-awareness enough to know that I've failed to "keep up the act" -- and I do feel like most of my life I've felt a pressure to not be myself because myself was "off" -- going by the reactions of other people, that is. Now my eye contact is very poor, I've noticed. I catch myself diverting my eyes and suddenly realize, many times a day, that I diverted my eyes at a moment when an NT would have maintained contact, or even that I failed completely to make eye contact, in a way that I used to make sure I did.

When I was younger, I was told that I tend to make too much eye contact. "Boring into and ripping out my soul" is how one person described it. Up until then, I always thought I had "good" eye contact LOL! Since then, eye contact makes me feel self conscious because I am never sure if I am doing it "right

For me it just feels extremely unnatural. As soon as I look into someone's eyes my mind just goes completely blank. I'm no longer listening to what they're saying and all I'm thinking about is the facial expressions I'm making to try and look like I'm a normal person who's actually engaged in what they're talking about

and a million questions whiz about through my mind... What do I do now? When do I look away? Is it going to get me into trouble if I avert my eyes? Am I in trouble? Why are they looking in my eyes? Do they think I'm lying and they are trying to "see" the truth? Are they trying to see if I'm not normal? Can they tell I am not normal? How can I get out of the room/area? When will they stop? Do I have to say something? Can I just look down at the ground? What about the wall? Can they tell what I'm thinking?

I tend to focus too much on it (which makes me stop listening) and it just feels scary to me.

Being stared at makes me terribly uncomfortable and self-conscious

...pulling your head down in embarrassment with desperate need to hide your eyes from others

Looking directly at people makes me feel embarrassed

When I accidentally make eye contact with strangers, I feel embarrassed and that I have to look away quickly because I've happened upon something that should remain private.

I use eye contact when I stare at a guy I like from across the room. Yet when I finally talk to them I make little to no eye contact out of nervousness and I experience a feeling of severe awkwardness.

I can't formulate thoughts properly while looking into eyes. It makes me flustered, as if I'm holding hands with them or something. It's embarrassingly personal

Whenever I accidentally make eye contact, I feel so embarrassed that I instantly look away, and if they are talking to me I usually get distracted and fidgety.

It feels really awkward and uncomfortable. I don't see why I need too look at people in the eye especially because if I do all I can think about is looking away which distracts me from what they are actually saying.

it's awkward and it doesn't feel natural

I find eye contact embarrassing unless I know the person/people well. It makes me feel flustered and I start blushing.

It makes me feel awkward and uncomfortable

Eye contact almost gives me a feeling of embarrassment, or just general discomfort... It's hard to explain. Everyone here summed it up pretty well I think. I even dislike looking at photos of people gazing directly at the camera because it's as if they're actually there, staring at me

It's not painful, just... awkward

At one time I used to get a feeling of embarrassment when making eye contact.

I get really embarrassed when I look into people’s eyes for too long?

**5) Nonverbal Communication**

***5.1 Difficulties Reading Information from the Eyes***

Sometimes I think I've "read" their faces correctly, but then I find out I didn't and feel stupid. Like I"ll see something in their shoulders or hear something in their voice - stress ? anger ? frustration ? at me ? or in general ? so I'll ask someone "is something wrong?" and they'll say no, so I'll be all "ok" and think it's true. Then I find out they were really mad at me and I'll feel stupid for not knowing it. and they're mad at me for not knowing it. Some people can "read" emotions in other's eyes but I just usually get it wrong. Like I'll think some guy is "making eyes" at me and flirting - and he's not. He's actually looking at me because he thinks I'm weird

Largely because when I just look at the eyes, I don't understand the emotion the other person is exhibiting. I need lots of other visual clues - posture, tilt of head, tone of voice. With eyes and mouth alone I really don't get it. It's like there is nothing there, emptyness, and that is very hard, it makes me very anxious

Do you have what is called the "direct-eye gaze"? I have that along with the inability to read tone-of-voice or facial expressions. Regretfully, I'm one of those guys who gives people the creeps. However, no one gives me the creeps, even with an ominous stare.

i can not be sure that NT's can not read my personality by looking into my eyes, but i am completely sure i can not read their "whatevers" by looking at their eyes

I don't get much emotional information from it, but it seems to keep people a bit more relaxed when they're talking with me.

I know that I have no trouble reading facial expressions when I actually CAN make eye contact normally, which is rarely ever

God, it seems so complicated and inane to me. Sometimes I wish people were more like books, able to express themselves well just using words. Some of the most "human" emotions I've had, when I've been able to feel the most empathy, was during a good book reading session. I try to read someone's face and I think "well, I guess I caught some of that but who the hell knows, it's all so random and it can mean so many different things". Yet when I read a great book it's like the author is laying him/her self bare, it can be just so utterly clear.

After that, I found myself staring (unnaturally I think) at others and we'd sort of get caught in this eye-lock thing and I never knew if they were thinking "Wow, This guy's really interested in what I'm saying!" or "What's that weirdo looking at?

My lack of eye-contact started off as the result of confusing social cues. I did not want to look at people who communicated very heavily with their eyes because it was difficult for me to understand

Meeting someone's stare and trying to analyze whatever message they're sending to me is almost nauseating at times.

I can see that people are using it to communicate something to me, but I can't tell what they're saying.

When I was a little kid (and I'm a very visual person) I used to get smacked for "staring." I got smacked for "staring" quite a lot, too. I don't think Mom realized that I wasn't intentionally staring at people and (in her perception) being rude, I was simply trying to read their expressions (which I am very poor at doing.)

As it is difficult to anticipate what will be communicated in a conversation or where it will go. I compensate for this, by focusing on a limited set of information being communicated (i.e. what is being said). As the other information coming in (say, from facial expressions, eye contact, body language) is too overwhelming to process quickly.

Eye contact doesn't come naturally to me, and I can't really read eyes anyway. I usually focus automatically on the mouth, even in videos and still images.

it's pointless, I get no extra data

As someone else put it, eye contact feels like looking deeper into each other's soul. For someone like me who is clueless to how others are feeling or what they are thinking, eye contact only betrays my confusion, makes me very selfconscious and that makes the interaction very awkward

I don't like feeling watched and eye contact and being filmed/having a photo taken of me feels that way. I think this is probably what's caused me to be unable to read faces.

In fact, there are some people, like my son, that I have no problem looking into his eyes...but others, like my husband, when I do I just see evil. I think for me, the eyes say a lot and I don't like what I see in most people's eyes.

I've learned how to read body language and facial expressions really well (I do great on all the tests)...but none of this translates to face-to-face contact. I lose access to that part of my brain. I rely as best I can on word choice and voice tone. And also theory of mind...I mean, seriously autistic children lag at developing it, but that doesn't mean we never get it.

Even when I did, I barely perceived emotion in them.

Yes that's a good description of it. I can pick up quite a lot of emotion cues from faces and bodies, but NOT if I have to hold a coherent conversation at the same time.

I think that's why when I took that emotional IQ test with pictures of people in different naturalistic settings showing different emotions, I was able to come up with elaborate scenarios of what everyone was thinking and feeling, but I didn't come up with the answers I was supposed to and ended up scoring below average.

Even if you don’t think you’re glaring, remember, we can’t read facial expressions very well and we can often think that you are.

Your eyes don’t talk to me, [your mouth] does.

But eye contact can be extremely difficult for people with autism, including me, and that’s because of a few different reasons. And one of those reasons is facial expressions. Facial expressions are extremely difficult to understand for people with autism. It can be extremely confusing like all the other forms of nonverbal communication

When you look at a person’s face, there’s so many details in a person’s face, so many things going on at the same time, so many tiny details of information about emotions, and about feelings, and uh expressions.

***5.2 Innacurate Nonverbal Sending***

…mostly peers, who interpreted my blank facial expression/lack of eye contact as either sadness or boredom with them.

I do (unconsciously) facial stuff that triggers off fight-or-flight responses in NTs too, which hasn't helped.

I can have a belated facial response to where is it consciously brought up, and the delay makes it appear disingenuous. It 'looks' like that you are searching for something.

I have a childhood packed with my parents saying "Stop staring", "Take that look off your face" etc. or conversly,

Strangely though, i don't feel intimidated, i worry about intimidating other people if anything.

If I get too overly anxious and focused, I stare into people eyes and look overly agressive

It is frustrating and I still havent learned how to look at people properly. I either have to look away or as I have recently been told, I stare and people find it intimidating.

I'm too busy measuring how long to maintain contact, attempting to look interested and engaged, that I don't have enough presence of mind left to actually be interested and engaged.

Eye contact never bothers me, as I can focus on their eyes and stare at them until they beg for mercy. Literally beg for mercy. I don't really see anything other than their eyes when I look at them and no 'connection' takes place, but I guess they get the connection that I am some wild sociopath that is trying to intimidate and ultimately crush them by staring them down.

This has been a real problem in my life, as you can probably surmise. My twenties was mostly being challenged by the 'tough girls' with me being the worse for wear because my 'challenge' was non existent and only in the mind of the beholder.

And generally when I do force myself to make eye contact I do so with such direct laser beam intensity that I either scare/creep the living hell out of the other person. Can't win for losing.

I think the eye contact we give can scare some people off. Maybe we have powerful eyes that can be intimidating? I know I hate intimidating eyes.

I think people pick up a discomfort vibe from a person via their expression or lack of smile and this makes them less likely to make the eye contact

Do you have what is called the "direct-eye gaze"? I have that along with the inability to read tone-of-voice or facial expressions. Regretfully, I'm one of those guys who gives people the creeps. However, no one gives me the creeps, even with an ominous stare.

I just envision most people who are not on the spectrum, having their eye motions in sync, being able to soften their gaze, add a sparkle and emotional richness - in direct response to how others react. Whereas my gaze may be cold and unemotional, unempathetic.

people sometimes hated me when I didn't make eye contact because they thought I was rude and didn't care about them (or respect them). When I made too much eye contact people were actually afraid of me

If people don't see my face, then I can't send them unintentional signals. Or at least that's the way it should work. So whenever I'm talking to people that know me, I try my absolute hardest to make sure they don't see my face

Maybe I lack confidence and worry it will bring too much confrontation into my life? People might misinterpret it as being ready for a fight and I might get into more scrapes this way, so I try not to look at people because of this.

On women, I feel if I look too long that I am flirting with them or hitting on them. And I feel awkward thinking that someone else thinks I am hitting on them. I guess the same can be true for guys. I don't want to feel like I hitting on some guy.

One eyebrow raised equates to "what the hell?" in most situations...which used to get me told off by certain adults because they would think I had an issue with something that they did or said because I can raise both eyebrows individually and would do so at random.

I'm kinda scared of looking into people's eyes because I think they will see me as a creep or a pervert.

My resting expression is such people get uncomfortable when I make eye contact. They think Im going to do something violent, It is partly because when I make eye contact I tend to over focus and thus look like Im giving them the evil eye

others may be misinterpreting my expressions...I just don't know

I cannot make eye contact when talking to people

be interpreted as showing disinterest (when it often isn't).

What blows me away is the number of NT's that still believe those who refrain from eye contact are all liars or crooks

What's horrible is that some people view lack of eye contact as a sign of dishonesty. I am generally a trust worthy person and do not deserve for people to be suspicious of me due to lack of eye contact. Being an aspie is tough especially when people do not understand Asperger's

For me it just feels extremely unnatural. As soon as I look into someone's eyes my mind just goes completely blank. I'm no longer listening to what they're saying and all I'm thinking about is the facial expressions I'm making to try and look like I'm a normal person who's actually engaged in what they're talking about

But eye contact with strangers who I needn't talk to is like one of the most hardest things to do, because:- -I'm afraid I might be intimidating them if I find myself staring at them too long -I'm afraid I might look silly if I try to make unnatural eye contact, where my eyes go all over the place and I look all nervous -I seem to have a fear of meeting a stranger's eye because then a voice comes into my head saying ''what if he/she's just looking at me because he/she thinks I look weird?'' when rationally they are just looking because it's a natural thing what people do -I then feel afraid that if I DON'T look at their eyes, they might look at me even more, expecting me to look at them -All in all, I just find making eye contact with random strangers feels awkward

-Some people think I have a weird stare and to them it can mean 'weird stare=time bomb waiting to explode' so then I get treated like a freak.

I realize it bugs other people when we don't do much eye contact because they may feel we really aren't listening if we aren't eye ball to eye ball, but if we really don't want to listen we can walk away or pick up something to read, etc. As long as we are there listening with our ears, we are participating in the conversation.

and a million questions whiz about through my mind... What do I do now? When do I look away? Is it going to get me into trouble if I avert my eyes? Am I in trouble? Why are they looking in my eyes? Do they think I'm lying and they are trying to "see" the truth? Are they trying to see if I'm not normal? Can they tell I am not normal? How can I get out of the room/area? When will they stop? Do I have to say something? Can I just look down at the ground? What about the wall? Can they tell what I'm thinking?

makes me anxious - am i staring too much and seem creepy? am i not making enough contact and seem "shifty"

-I feel like they are getting some kind of wrong impression from it

Much like others have said, I don't do much eye contact because it doesn't come naturally to me. I don't really know the appropriate timing, and I feel at risk of staring for too long, which could be taken as a threat-stare or a sexual stare.

If I **don’t** participate in, to me, this ext**reme**ly inappropriate intimate act of direct eye-gazing for long periods of time, people are going to tell me either I am shy, submissive, or I’m being rude by looking away. **Or** they would assume I’m being shifty by looking away and I don’t know why that is.

in this world people seem to think that if you don’t make eye contact you are **not** listening and if you don’t make eye contact you’re being dishonest [pause] so this can this can make it very difficult to people with aspergers because as soon as you meet somebody who doesn’t know you have aspergers and doesn’t understand the difficulty of it they think you are being dishonest and you’re not listening therefore you’re rude and a liar.

**6) Society & Culture**

***6.1 Importance***

eye contact sucks, but i guess we have to work on it

The main reason is that people expect it. Not being able to make eye contact can cause social difficulties

Eye contact, remembering people's names, remembering people's histories, remembering to respond to requests, remembering what things a person can be expected to know or not to know, guestimating who might know whom... All annoying, tricky, frustrating, and essentially mandatory tasks which one must find mechanisms to emulate and which one ignores at one's peril.

Because it's useful to have people think that I'm honest and interested in what they're saying. It makes life run more smoothly

When I was very young, I listened much better when I didn't have to look at the person.

When I became older I realized other people estimated ones position in a group by the level of confidence that person perceived from them, and NT's associated lack of eye contact with lack of confidence, and unfriendliness. I realized I was mis-representing myself so I practiced making eye contact by staring at photos of people online.

Eye contact is a must have on a conversation or interaction between two people. It is an acknowledgment. I am here I listen to you I understand you I agree/disagree

But whenever I'm around people I don't know, I force myself to look at their face just so I don't appear to be any weirder than I already am

i do actually try to make eye contact because i know it reads badly to other people if you do not

The thing about eye contact is it's important to make some eye contact, but not too much

I do enough eye contact to show respect and that I'm listening to the person

To put it bluntly, I suck it up and just deal with it. Noone goes through life without experiencing discomfort or unappealing things

It is a good skill to learn when you job interview. Eye contact is important. There are ways to get around it when you have to

Eye contact is one of the most uncomfortable things in the world to me... And because I work in sales I have to do it a lot and I F***ING HATE IT!

It kills me to do it, but it's what I have to do to get by in this NT world.

And eye-contact feels rather pointless to me, though intellectually I suppose I could pick up something about the person's mood if I occasionally glanced at their face.

Some people are like, “oh how rude he’s not making eye contact with me.” It’s not that I’m being rude. It’s my disability, but it’s still no excuse you have to make it an effort.

But, I do think it it’s an important thing to sort of learn to face

After a few years and I was about sixteen. I just was like. I want to be able to look people in the eye, I want them to know that I’m not being rude. I’m being respectful and I care about what they are trying to say

the other thing I found difficult is I'm because I'm now aware that it’s important for some bizarre reason

so yeah I'm just really not sure... how to advise anyone who’s having this difficulty all I can reassure you is that I have the same difficulty and that [sigh] it is a big problem in my life that I don’t think I’ll be able to overcome

Eye contact is a quite an important thing in our society. When you’re having a conversation with somebody, this person expects you to make eye contact.

***6.2 Lack of Importance***

I never understood the need for it. People can hear me just fine, and I can hear them without having to look into their eyes. I've no interest in looking people in the eyes just because it's some unspoken social rule. I also suffer from a strong "look away" instinct if my eyes accidentally meet someone else's

It makes me uncomfortable and I never saw the point in it.

It just doesn't make sense. Why would you want someone to look into your eyes? Unless maybe your in love with them but otherwise...

peoples eyes never change shape or color. they never flex or bend in concert with the flow of conversation (optical gesticulation (makes me sick to think about)), and i think it is useless to look at eyes because they are just organs of sight.

If I **don’t** participate in, to me, this ext**reme**ly inappropriate intimate act of direct eye-gazing for long periods of time, people are going to tell me either I am shy, submissive, or I’m being rude by looking away. **Or** they would assume I’m being shifty by looking away and I don’t know why that is.

I often find that people seem to **like** you to make eye contact which is kind of another bizarre thing.

***6.3. Neurodiversity Advocacy***

It's that eye contact, facial response loop, that we aspies lack, that cements the social web into place for neurotypicals. We've had a life time of getting negative responses to our performance against that scale - it's no wonder eye contact upsets us

My mother used to force me to look her in the eye, but usually it was if she thought I was being dishonest. The same with my teachers. I also have low self esteem, am anxious, and generally don't like confrontation. So I have always associated looking one in the eye as being a case of being ridiculed, questioned or doubted

I don't want to force myself to do something just to please others

NTs seem to think that their assumption that lack of eye contact means that someone is disrespectful, untruthful or are hiding something is the only thing that it means

It can be about as comfortable as standing naked outside in zero degree weather near the entrance of a very busy shopping mall. I hope that helps. I'm saying this for the autistic three-yearold girls of the world who can't articulate those feelings

The need to force others to look people in the eye seems to be deeply embedded in human culture. Even with my excuse, I did get some pressure. But it is absurd, prejudicial, and unfair. I am, to some extent, physically unable to do so - and I was made to suffer for that. You might argue, in that case, it is better to force children to learn to do what will spare them suffering. But, this is also how we are wired. I argue that, instead, the bigots need to stop forcing us to be like them, and accept who and what we are. The thought of your colleague lecturing you to perpetuate this tyranny (yes, I consider it harsh enough treatment that tyranny is an apt word for it) infuriates me.

I think there should be a lot more emphasis on helping an autistic person to be as comfortable as possible in the world. Discomfort and stress make autistic traits much worse, and the opposite is also true. The more comfortable and relaxed an autistic person is, the better they can function. So they need to be allowed the freedom to be who they are, and not be taught behavior that will make the NTs around them more comfortable at the expense of their own wellbeing.

I can only speak for myself, but I would prefer a focus on educating NTs rather than trying to create NT-like behaviour in those on the spectrum. Most of the higher functioning members of the autism spectrum 'club' will learn how to fake it in public with or without assistance. The trouble is, no matter how good we get at fitting in, it is never sustainable.

Forcing an eye contact constitute an abuse, It similar to raping a child. If anyone of the so called pro's where willing to pay a little attention and make the effort to infer from the many known AS traits an identity the reasons behind the way AS managing their eye contact would be quite clear but since the pro's approach is based on assuming that anything we do or say is a symptom of a disease or malfunction no one would bother.

So you train children to focus their eyes on the person talking, you try to postpone the discharge reflex as late as you can. The basic assumption is that by fixing that eye contact it will somehow bend the aspie towards this "acknowledgment". And you came to this forum to challenge this long shot assumption. Are you really working on the awareness of the autistic child? or are you just making a cosmetic change in order the child to be socially acceptable?

Never the less, I don't think you should be forcing anyone on the spectrum to do anything they don't want to do - especially if it causes distress and pain for them - just for the sake of making sure they fake being an NT

I think it can take away focus on helping children with REAL necessary skills

i am expected to do something that makes ME mentally/emtionally uncomfortable so that OTHER PEOPLE feel better. and if you 'train' and aspie or autie to do more eye contact, you will still have no assurance they care or understand what you are talking. so there is truly not much point, except to keep up false appearances. they can learn it themselves when they are older if they want.

also I don't want to fit in with the normals and their way of living.

I don't understand why it matters, and I refuse to get upset over something that seems so pointless to me. If other people have a problem with my level of eye contact, it's their problem not mine

Very recently though I have found how much less anxiety I experience throughout the day when I do allow myself to consciously avoid eye contact; so I am doing more of it. Rather than buying in to the notion that I should be more of a neurotypical extrovert and make lots of eye contact, I am enjoying allowing myself to just be me, and only make eye contact when it serves a good purpose. The anxiety-reducing effects of this new approach are, so far, one of the benefits I most enjoy about being newly diagnosed and having a better understanding of myself.

Advice on how to maintain eye contact? Personally I don't see why we should have to. It's ridiculous, and I don't.

I always think people are staring to judge, which I always thought was wrong, especially if you're not doing anything to attract such stares, which I know I don't.

But I suppose it's another double standard. It's ''OK'' if NTs do it but an Aspie is always told it's socially unacceptable to do is and so ''mustn't'' do it.

I became aware quite a few years ago that eye-contact was something of an issue with me, and went through a phase of trying to do something about it, in situations where I felt it might be required. I don't think it was very successful - it always felt very artificial , and I had the impression that the recipient was at least as uncomfortable with it as I was. These days I just don't bother. My default reaction is always immediately to avert my gaze in these situations. After four decades of trying, and generally failing, to adapt to the social, cultural and attitudinal expectations of society in my adult life, I am quite happy to be a misfit

just when I think this issue is moot someone brings it up again - I was at a group recently where someone who was a recovering drug addict said something along the lines of "I was finally able to look people in the eye again," about her recovery from addiction. I really don't understand why this is still so black and white in multicultural western countries. From her comment, a lack of eye contact still indicated shame, an inferiority complex or fear. Good eye contact indicated self-worth, strength and honesty. It's SO idiotic - don't people even realise there are a load of other possibilities out there, ranging from autistics to native peoples to alternate cultural norms???

The whole business reeks of trying to fit in with a style that just isn't me, which seems kind of undignified

Some people are like, “oh how rude he’s not making eye contact with me.” It’s not that I’m being rude. It’s my disability, but it’s still no excuse you have to make it an effort.

Just because we’re not looking at you, doesn’t mean we’re not listening,

for people to demand ‘look at me’ or ‘stop day dreaming and listen!’ is not just insulting to us but it’s demeaning and upsetting. This has to stop as its little more than an attempt to change us and make us behave in a normal way.

If I **don’t** participate in, to me, this ext**reme**ly inappropriate intimate act of direct eye-gazing for long periods of time, people are going to tell me either I am shy, submissive, or I’m being rude by looking away. **Or**  they would assume I’m being shifty by looking away and I don’t know why that is.

in this world people seem to think that if you don’t make eye contact you are **not** listening and if you don’t make eye contact you’re being dishonest [pause] so this can this can make it very difficult to people with aspergers because as soon as you meet somebody who doesn’t know you have aspergers and doesn’t understand the difficulty of it they think you are being dishonest and you’re not listening therefore you’re rude and a liar.

if you have any other sort of advice on how to do this apparently critical thing in the neuro-typical world this kind of social acceptance of making eye contact then leave a comment

**7) Strategies**

***7.1. Exposure & Practice***

I try to force myself for very short burst to look straight at people.

I used to draw people's faces a lot, out on a bus, train, or coffee shop. from the side, or someone too far away to realize what I was doing. And i think when I was doing this, I was a lot better at looking people in the face during discourse.

i try to stare at mt cats as an exercise

Practice on someone you feel the most comfortable with - even a relative. You may not be able to maintain it and it can be scary at first. Try for a few seconds at a time. Once you can do that,

practice on other people

Use a mirror to practice with yourself.

Just remember that you don't have to maintain eye contact the whole time, just on and off otherwise you look crazed. It ain't easy but you can do it if you practice.

I had a close family friend help me overcome it because in her culture eye contact is very important. She encourages my siblings who are NTs but a little on the shy side to make eye contact too.

try looking at yourself in a mirror in a really attentive way with your eyebrows raised and your eyes slightly widened. Then try relaxing your eyes and your face totally and just staring back at yourself blankly. Don't worry about the facial expression, just look at your eyes. You'll see the difference between eyes that are 'alive' and eyes that are 'disconnected'. The trouble is that, because some autistic people need to unplug their brains from visual input whilst they're listening to something, they give out the disconnected eyes/autistic gaze thing without realising it.

you could try talking to those people that you intimidated and try to explain it to them, so that maybe you could practice to get better.

I practiced making eye contact by staring at photos of people online

If it is generally easier to make eye contact with animals than with humans, that might be a less distressing first step and making eye contact with humans might be the next step.

It's one of those things that you just can't give/receive advice on, you just have to practice it.

It's always a trial of determination and practice caked in discomfort, but it almost always pays off.

Practice in a mirror so you get comfortable with the feeling of doing it and your body language is more relaxed.

So the best advice and I give people who want to make it an effort to make eye contact is to practice doing it.

Whenever I’m in a situation where I have to give someone eye contact, for example if I’m one on one with someone or in a serious conversation with someone, I force myself (points finger to forehead) to do it even if it lasts me two seconds I’m still making it an effort.

What really helped me was my speech therapist. She worked with me on eye contact and I actually got some improvement and I made more eye contact, but even though I was making eye contact, I did not do it on a consistent basis.

I kind of trained myself, I just kept trying. First, you have to practice on your family. And you just keep staring at them in the eye until they. Until it just becomes natural.

It doesn’t really come naturally it becomes easier I guess.

You guys probably it feels super hard for you but just take baby steps.

Try it on people that are close to you and then try it on srangers and keep practicing until you get it.

***7.2. Barrier***

Dark glasses may be the answer.

It's easier to make eye contact with sunglasses on. When I know they can see me making eye contact, it's harder to do it.

perhaps try wearing sunglasses while you practice

I overcame the eye contact thing by wearing irlen contact lenses

Try talking to someone with shades on.

As an adult I've taught myself to make eye contact when talking to people but it's a very conscious effort. If I do it fast and look away I can usually maintain my train of thought. I've also reverted back to wearing glasses instead of contact lenses. This gives me a little bit of a barrier to hide behind. Somehow eye contact through the lenses isn't as difficult as having "naked" eyes.

The best part of wearing glasses is they give me a little something to hide behind. A lot of times the glare from the lenses hides the fact that I'm not looking you in the eye

I wear sunglasses. Besides my being ludicrously sensitive to bright light... this pretty much averts the whole problem of eye-contact. Naturally, people have their own twisted ideas of me due to my always wearing sunglasses in public... but since they never voice those opinions to me, I tend not to give a toss. Seriously. I could never go back to NOT wearing sunglasses.

you’re about to learn a major secret of mine. You see these glasses here, they’re not ummm as you can see they don’t magnify or anything. These are just, these are pretty much just typical glasses, they’re not reading glasses, there’s nothing special about them. I how they sold me is that wearing these glasses it’s like I have a movie screen in front of me so it’s like I make eye contact but I’m more, it’s more distance, you know like I can look at a movie screen and see the characters eyes and everything. And so it’s far more effective. And so the eye glasses are perhaps the biggest thing that really helped me with eye contact. Because you know there’s this extra barrier.

***7.3. Observation***

I also have tried to watch other people to see if I can figure out how they do it, but for some reason, I can't figure out which eye they are looking at, or if they are switching back and forth, or what.

Another thing you want to work on is observation. You want to notice what other people do what people who umm seem to have not make other people uncomfortable with their eye contact do and then you can try to mimic that.

but when they say eye contact they mean "keeping an eye on the muscles surrounding the eyes so as to know how the person is feeling", they don't really stay pupil to pupil as we tend to do when we learn eye contact. i think we were all mislead by the "look me in the eye"we got from day 1. that's, once again, not what they mean.

People seem to spend more time looking at the left eye, so that's what I do

***7.4. Counting***

Now I follow the 3 second rule (e.g. don't stare at someone for longer than 3 seconds, and try to look at them at least once in every 3 seconds if you aren't already looking at them)

I've had to make sure I maintain eye contact for the appropriate length of time. The average American maintains eye contact an average of 3-5sec (may be different in other countries). I tap my toes (not my foot) to count out the seconds. If I get to five sec, I take a fleeting glance at the wall or at my toes. No one has accused me of staring for months now.

be sure to look away (away and up, never down) every 2 seconds or so; otherwise you'll be staring aggressively, and being seen as aggressive is even more problematic than being seen as furtive.

***7.5. Mental Distraction***

One thing, if you can desensatize yourself mentally to a persons eyes, you can look at them much similarly to how you might look at an object

I generally just stare at the person's eyes as an object. I don't see anything when looking into them but color.

You just need to develop some mental filters that stop you actually seeing the person you're smiling and eyeballing

But now I've trained myself to look at the coloured flecks in people's eyes. It's not quite the same as making eye contact because you're using your eyes for looking and not communicating, and people can always tell the difference – but I've found that I can at least pay some attention to what a person is saying if I just find something in their eyes to focus on. And it makes you look less submissive and more interested, even though most people can tell you're faking it!

Keeping up another train of thought is probably the best way to do eye contact without distress.

You don't have to force eye contact. That's going to set you back quite a lot in terms of cognitive functioning. FAKE it instead. Takes some thinking about at first

I now know one of the important things is not to force yourself to have eye contact and not to think about eye contact. If you think about eye contact, that makes you very self-conscious and awkward, which makes the other person very uncomfortable as well. I realize good eye contact happens rather naturally without being too conscious of it.

One that has not been listed is to pick a salient feature of one eye and track that.

***7.6. Motivation***

No matter how bad you are at it, give yourself a reason to do it. Like you want to show you're paying attention, or you want to try to read someone's expressions. Learning to read people helped me a lot since I play poker. Even though it doesn't come across as friendly.

My advice is to learn about showmanship. What helped me learn was I used to be a member of the International Brotherhood of Magicians and would listen to professional magicians give advice at meetings.

Now do you wanna know what really helped me with eye contact? Dating. Ummm because of dating and you know that has helped me more than anything else. And what ended up happening, is that I went on one date a few years ago. Oh it was Valentine’s day the only Valentine’s day I ever had in my life, and ummmm and then…I then during dinner I didn’t make any eye contact whatsoever. Annnd, I was very embarrassed by that because I could make occasional eye contact but you know just I was really nervous and everything and didn’t make any eye contact

***7.7. Other***

I look out the corners of my eyes, in that cute little way.

PICK ONE EYE.

**8) To Compensate for lack of Eye Contact**

***8.1. Non-eye Fixation***

I just look at mouths.

My advice for if eye contact intimidates or scares you like it does me is just look at the whole face. If you look at it as a whole you don't find it scary (I don't at least) and it looks like you are making eye contact.

I look at their eye for like a milisecond and then glance to the side, then back to their eyes again, and then glance at their nose or something.

Usually I'll look at people's mouth.

I always look at the mouth, but I generally look at the floor or at what I'm doing rather than look at people.

Yeah, I remember learning something similar as a teen, which helped a lot. Looking from the eyes, to the mouth, to the gesticulation, etc.

The good thing about looking at the conversation partner's mouth is that they often don't even see where you are looking, and think that you're still making eye contact.

I tend to look at peoples mouths when I'm speaking to them if I have to focus on them rather than something I'm talking about. I find that it not only helps with avoiding eye contact but it also helps me follow what they are saying better because I lip-read a bit and I can see what they are saying as well as just listening to it.

I look at people's hair... I feel it's the least scary part.

I also look at the bridge of their nose, or their mouth.

yeah i usually look at their mouths, especially if they're talking and it's moving. if i have to make eye-contact with someone i know i look at the bags under their eyes, and then i slide up to meet their eyes and then slide down to eyebags again. i don't think people notice it so much if you look somewhere really really close to their eyes.

I realized that most people make at least some sort of gesture with their hands while talking,so i follow their hands while they speak. That way everyone thinks i am extremely focused on their discussion and doesn't complain about me not looking at them directly.

mostly I give pseudo contact by looking at the person's mouth. If we're talking about a specific object, such as a piano, I'll keep my eyes focused on the object in question.

Making eye contact... it is really more about looking at someones face. And maintaining it is about looking at different parts of their face and doing what the others said about leaning in and smiling. Although your actual eyes will meet with theirs as you look at their face it is not so much about looking right in the eyeballs.

where I usually keep my eyes during conversation: the shoulders.

If a person is particularly intimidating, I look at things near their eyes, such as their lower forehead or nasal bridge.

Now, I tend to look at eyebrows or noses, with a short glance at eyes of familiar, trusted people.

I prefer looking at their mouths because it's much more helpful for me.

ive been told i have goodeye contact.. but what they dont know is im looking at their nose

look at the person between the eyes- it will seem like you are doing eye contact even when you are not.

Just glance at the person's forehead when you or they get to the end of a sentence. It usually seems to be enough to make them feel that eye contact connection and is still short enough to keep me from getting overloaded. It takes a bit of practice but it's doable.

No, I've just learnt to substitute it; fake eye contact by looking at a persons nose.

Here if you can't look at my eyes, try looking at my mouth or forehead. Thats what I do.

I try to look at their nose or eyebrow or cheekbones or somewhere in the vicinity of the eyes.

I assume you’ve heard you don’t even have to look at the bridge of a person’s nose, you can look at their mouth, you can look at their forhead, you can find pretty much anywhere in the vicinity of somebody’s head, you can even look at their neck, and mostly just you know just don’t be too obvious about it. So that’s one thing you can do just sort of find some place in the vicinity of their eyes of their face that you can stand to look at.

I always just look at people's mouth's when they talk and try to look at their eyes every now and then when I talk.

I tend to look at or near them rather than make direct eye contact too much. I might look at their hair, eyebrows, nose, mouth, chin, lips or other part of the face, briefly meeting their gaze to acknowledge, but not staying there.

My best advice is you don't look directly into the eyes. Look at a point above the eyes

What I do is to look in the direction of their eyes, without actually focusing on their eyes.

You don't need to look at their eyes. Just look at their bodies. But if it's a woman, don't look at their chest. Or you can look towards them.

I still only use brow/mouth/ 30 degree alteration contacts

When I'm trying to process what others are saying to me I find it most helpful to watch their mouths

my father taught me was how I could look at the person's forehead or even just above their head, and from a distance of even a few feet they would not be able to tell that I was not looking at their eyes

I look at people's mouths also (if I am looking at them at all). It seems less rude / dodgy than not looking at their faces, and also helps me make sense of what they're saying.

Look at the person's nose for the duration of the conversation. They'll believe that you're making eye contact

I stare at the area in between the eyes.

You can look directly into one eye for 2 seconds, then look elsewhere on their person, like their forehead or hair for several seconds, say 5, then come back to the eye for a couple seconds, then look off at an object for maybe 10 seconds, and so on.

Just look at their nose or forehead.

I trained myself to look on their noses so they won't feel like I'm weird or not interested but that's also pretty exhausting.

I usually look at their mouths.

i look in between their eyes.

I generally look at their mouth. It's much easier than staring at one/both eyes, but it gives NTs the "respect" they crave through body language.

I tend to look at people's mouths and sometimes their nose

I usually focus on their mouth.

In an interview change eyes to eyebrows and nose and make sure you spend some time looking in those areas and you will be fine.

So I usually just look at the area between nose and mouth whenever I feel like eye contact is expected of me.

usually i make nose, teeth or fronthead contact.

Eyebrows and temples. A therapist taught me this in 4th grade

I usually do mouth and a little eye contact

I just look in their general direction.

Eyebrow and nose contact are the best. Nobody has ever commented that I have weird eye contact so it must work

I sometimes look at their nose or eyebrows.

I tend to look people between the eyebrows

I stare right at the bridge of my boss' nose until the helpful conversation is over.

I work with looking at the mouth with an occasional very brief flick up to the eyes when I remember and to see if the person is paying attention

I usually just watch peoples' mouths or a point between their shoulder and their ear.

I just look in the general direction of their face, without focusing on eyes.

Part of a way of faking eye contact is to look at there nose or mouth or maybe a skin blemish

Looking at someone's mouth is completely fine - I know people who do this and it looks like they are making perfect eye contact. Looking at the nose is a good idea too

I just run my eyes over the person I'm talking to every once in a while, and look in the general direction of their head while conversing.

I try to look at a spot on the face that will make it appear like eye contact, and incorporate body and head movements that allows me to look away regularly.

I practiced looking at the spot closest to the eyes that I could manage: first I could only manage the collar or hairline, then I worked my way in to the chin or forehead, then the mouth or eyebrows.

So what I would usually do is I will look away for a while and I might look at your shoulder or I might look at your hair

If someone is trying to make eye contact and I feel uncomfortable I just look them right between the eyes. Peolpe dont notice and I can relax and consentrate on the conversation

but if you just change things up you know honestly look at somebody’s nose and then look away and then look at their eyebrows and then look away

I’ve heard about the idea of looking at somebody’s nose rather than their eyes but that still creates a huge problem because it’s still concentrating on something I still have to look at their nose. It doesn’t make me as uncomfortable I find it a lot less uncomfortable to look at someone’s nose if I looked at someone’s eyes that makes me very uncomfortable. But if I just look at their nose it’s less uncomfortable but I'm still not listening.

***8.2. Verbal Backchanneling***

I try my best to counteract this by practicing active listening skills. For example I'll paraphrase what the person said. This lets the person know that I was paying attention, even if I didn't look attentive.

I would also say stuff like: really, sweet, I know what you mean, thats cool, no s**t, stuff like that to show I am following their sentence.

I also do little split second eye contacts before looking upwards and saying hmm, with a nod.

***8.3. Nonverbal Backchanneling***

when I'm not doing eye contact I make sure to do other listening cues

Before I knew I was autistic I would nod my head as the person was talking to let them know I was still listening

try to compromise by doing lots of appropriate body language, not crossing my arms, smiling and making "active listening" type noises to show I'm listening. It can look attentive, pensive and mindful as long as you respond with pertinent questions and don't suddenly pick up a rubiks cube while they're chatting. People want to know you care; and if you show that then it shouldn't have to be as didactic as percentage of time looking as opposed to not.

I think the reactive cues such as nodding, and saying "mm hmm" and so forth when the other person is talking are easier (though also difficult and distracting) to include to show somebody you are paying attention than learning how to look at their face at the appropriate times

Try to give other cues that you're listening intently, like leaning forward slightly, nodding at the right time (but not too much!) etc. Worrying about it just makes you look more nervous and gives the wrong impression.

smile is a gesture of friendliness, confidence, assurance that you are enjoying their company and conversation and paying attention to what they say, and makes that very small amount of eye contact count enough and convey enough of what they are looking for that you can carry quite a long conversation on just a few of them and the NT person will probably be perfectly happy.

I shake my head a little bit (begins to nod head to demonstrate), oh yeah, uh-huh, you know, respond to them (continues to nod head to indicate listening to conversation partner). That’s the best way to show people you care, you’re paying attention to what they have to say.

Um, my eye contact is strange but I’ve worked at it really hard and I have a few tips that I think will um might help you if it’s something that’s difficult to you. One of them is simply to look up rather than looking down*.* And I mean you don’t have to look up at the eye, no just know keep your head on an even level and you don’t have to turn your face towards the person who’s speaking. You can have your face turned just a little bit away, and for me at least that’s significantly more comfortable but it’s less um odd to other people then looking down*.*

just sort of nod and don’t even have to look at them, just do something that indicates you’re engaged with them, nodding and not looking works really well for me.

Try to give other cues that you're listening intently, like leaning forward slightly, nodding at the right time (but not too much!) etc. Worrying about it just makes you look more nervous and gives the wrong impression.

***8.4. Disclosure***

Sometimes I'll tell people I'm still paying attention even though my eyes are wandering.

And if I ever get called out on it, I either say "yeah, my ears are blocked at the moment, so I have to look at your mouth" or "I'm the kind of person who needs to look away a little to really concentrate on what you're saying." The latter is courtesy of Tony Attwood. It all works, you've just got to come across as confident.

But yeah I think if someone questions me about my lack of it I'll just be honest. I'll just say 'It's hard for me to listen and follow the conversation if I try and make eye contact, but I am listening.' If someone cant accept that I think they have an issue I mean its not like their health depends on my giving them eye contact.

***8.5. Body Position***

How do I avoid it? Simple; don't sit in front of someone you are talking to. Sit beside them, behind them, or anywhere other than face to face. If you're in a car going somewhere with someone this is easy, as one of you is watching the road, the other doesn't expect to have eye contact. Otherwise sit on a couch, armchair, or bench or whatever. That's why it's great going to the movies, watching TV, or going to watch sports; you have something else to keep your eyes on and you aren't considered to be odd by not maintaining eye contact.

my actual preference for conversation is "side by side" and looking out

***8.6. Blurred Focus***

I not sure how to describe this, but I also unfocus my eyes, so I am giving eye contact, but for me its a fuzzy, less intense view, sort of like gazing.

I've come to realize that I don't make eye contact with people. I just look through them.

one eye contact alternative I've used before is to "look through" someone, to focus my eyes on a point behind their head.

I do the looking through the person to the back of their head thing and glazing my eyes over so my vision is blurry. I'm sure they can't see my blurry vision.

I cross my eyes a little so their eyes become blurry to me

Ever since childhood I have got used to unfocusing my eyes and looking just between their eyes. It's like wearing clothes and not staring into windows. It works for me

I really don't have a choice in eye contact or not, when I look at a person I can't hear what their saying, sometimes I look in their general direction but "turn off" my eyes, I don't close them I just only focus on their voice, or I turn my good ear to them.

I now look at the face, but do not focus.

***8.7. Strategic Eye Contact***

I've found that eye contact is essential when starting a conversation with someone. Once the ball gets rolling, eye contact is not required that much anymore. That's when I do it in short bursts, every once in a while.

When I want to seem neurosocial, I make eye contact for 1 second from time to time. Just enough to not appear evasive.

Have you tried just making quick "glances" at the person you're talking to? I've found that that is easier to maintain a conversation if I'm not staring intently at them and instead looking away every so often. This helps me prevent that feeling of being overwhelmed.

When it's my turn to speak, I give fleeting eye contact.

If they're looking at you, you make eye contact for maybe 1 second, smile SLIGHTLY, then look away.

If it's during an interview or something like I had recently (and apparently did extremely well with)…I make eye contact every time I start an answer.

I find if you just glance at them periodically, so they know you haven't tuned them out, they're generally happy with that, but I tend to stare at a spot on the ground just behind and beyond the speaker.

My tip is to look when you can, even just glances

I started to just glance, as though "scanning" the mirrors in a car while driving. Maybe once every five seconds or so.

I usually glance at them them look away and then make sure I kept having the occasional glance so they know I'm interested.

What I do is actually look a little to the side so it looks like I'm looking them in the eye, maintain that for a few seconds, look away, repeat after a little while.

I will have quick glances at their eyes, preferably when they are not looking directly at me.

I just look at their nose and let my eyes unfocus.

The best I've come up with is the occasional glance to reassure the speaker that I'm listening

I usually just take a quick look at the general face area and then look away

like when someone pauses then I look at them and it appears they were waiting for some eye contact acknowledgement so I'll glance up just to give indication I am listening and then continue on

I kinda look back to their eyes and then keep looking away again, as if I'm thinking.

another thing that actually I do a lot is I look at somebody uh and then I look away, and then listen to what they’re saying.

I look at them when they’re when they start speaking, I look away while they’re speaking, I do something maybe with my hands
